# Revision of the genera of Heligmonellidae (Nematoda, Heligmosomoidea), parasitic in Muridae from New Guinea[Fn FN1]

**DOI:** 10.1051/parasite/2023058

**Published:** 2023-12-20

**Authors:** Marie-Claude Durette-Desset, María Celina Digiani

**Affiliations:** 1 Institut de Systématique, Évolution, Biodiversité (ISYEB), Muséum National d’Histoire Naturelle, CNRS, Sorbonne Université, EPHE, Université des Antilles 57 rue Cuvier, CP 51 75005 Paris France; 2 CONICET-Consejo Nacional de Investigaciones Científicas y Técnicas Argentina; 3 División Zoología Invertebrados, Facultad de Ciencias Naturales y Museo, Universidad Nacional de La Plata Paseo del Bosque s/n 1900 La Plata Argentina

**Keywords:** Nippostrongylinae, Synlophe, Rodents, New Guinea

## Abstract

Up to now, 24 genera of Nematoda belonging to the Nippostrongylinae (Heligmonellidae) have been reported from New Guinean murid rodents. Nine of these genera have been reviewed in previous works. In the present work, another 11 genera are re-examined on morphological characters mainly corresponding to the synlophe and to a lesser degree to the bursa. This re-examination leads us to recognize three valid genera: *Melomystrongylus*, *Pogonomystrongylus* and *Nugininema*. The remaining genera appear to us insufficiently described or seem to involve more than one taxon; we consider them *genera inquirenda*. These are: *Mawsonema*, *Montistrongylus*, *Parvinema*, *Missimstrongylus*, *Flannerystrongylus*, *Helgenema* and *Paramelomystrongylus*. The genus *Rodentanema* does not belong to the Nippostrongylinae but to the Herpetostrongylidae (Heligmosomoidea). In addition to the three genera recognized herein, nine other genera of Nippostrongylinae are present in New Guinea: *Equilophos*, *Hasanuddinia*, *Hasegawanema*, *Hughjonestrongylus*, *Lesleyella*, *Macrostrongylus*, *Nippostrongylus*, *Parasabanema* and *Sanduanensis*. Several species attributed to the genera *Bunomystrongylus*, *Chisholmia*, *Odilia* and *Sabanema* are insufficiently described and their generic assignment could not be rectified or ratified. Consequently, the presence of these latter genera in New Guinean rodents remains unconfirmed, until more complete descriptions or illustrations are provided.

## Abstract (fr)

Jusqu’à présent, 24 genres de Nématodes appartenant aux Nippostrongylinae (Heligmonellidae) ont été signalés chez des Rongeurs Muridae de Nouvelle Guinée. Parmi ces genres, neuf ont été révisés dans des articles précédents. Dans cet article, 11 autres genres sont réexaminés sur des caractères morphologiques concernant principalement le synlophe et en moindre mesure, la bourse caudale. Trois genres sont considérés comme valides : *Melomystrongylus*, *Pogonomystrongylus* et *Nugininema*. Les genres restants nous paraissent insuffisamment décrits ou représentent plus d’un taxon ; ils sont considérés *genera inquirenda*. Il s’agit de : *Mawsonema*, *Montistrongylus*, *Parvinema*, *Missimstrongylus*, *Flannerystrongylus*, *Helgenema* et *Paramelomystrongylus*. Le genre *Rodentanema* n’appartient pas aux Nippostrongylinae mais aux Herpetostrongylidae (Heligmosomoidea). En plus des trois genres ici reconnus, neuf autres genres de Nippostrongylinae sont présents en Nouvelle Guinée : *Equilophos*, *Hasanuddinia*, *Hasegawanema*, *Hughjonestrongylus*, *Lesleyella*, *Macrostrongylus*, *Nippostrongylus*, *Parasabanema* et *Sanduanensis*. Plusieurs espèces attribuées aux genres *Bunomystrongylus*, *Chisholmia*, *Odilia* et *Sabanema* sont insuffisamment décrites et leur assignation au niveau générique n’a pas pu être rectifiée ou ratifiée. Jusqu’à ce que des descriptions plus complètes soient apportées, le statut taxonomique de ces derniers genres chez des Rongeurs de Nouvelle Guinée reste incertain.

## 1 Introduction

New Guinean rodent fauna is considered one of the most speciose in the world [11]. All native New Guinean rodents belong to the Murinae, a large subfamily with origins in Southeast Asia [20]. Several of these native species belong to the genus *Rattus* and are thought to be recent colonists (the “new endemics”), arriving about 1 my ago, whereas the remaining species comprise a group of “old endemics”, arriving about 5–10 my ago. These old endemics seem to be monophyletic, resulting from a single colonization event from which subsequent and rapid diversification followed [22, 34]. Rodent colonists from Southeast Asia to the Sahul region, as well as those involved in multiple dispersal events between Australia and New Guinea related to successive sea level fluctuations [22], probably carried with them their communities of helminth parasites, as noted by Smales [34].

Studies on the helminthological fauna of New Guinean murids were scarce up to the 1990s with very few reports published before 2000 [34]. It is from 2001 on that a substantial amount of information has been produced, mainly with the considerable work carried out by Smales [34–44], Smales & Spratt [46, 47] and Smales & Heinrich [45] through the publication of a series of papers on the gastrointestinal helminths of about 30 species of indigenous murid species.

Between 2008 and 2021, a little fewer than 20 articles were published concerning the helminth fauna of 26 of these species. These works evidenced extraordinary diversity of helminths comparable with that of their hosts. These reports and descriptions of new taxa concern mainly species of Nematoda, among which trichostrongylins are the most numerous; most of them belong to the cosmopolitan subfamily of the Nippostrongylinae (Heligmosomoidea, Heligmonellidae).

In these articles, 12 new genera including 18 species were erected (Table 1): *Melomystrongylus* Smales, 2009 with two species [39, 45]; *Mawsonema* Smales & Heinrich, 2010; with one species [45], *Montistrongylus* Smales & Heinrich, 2010 with four species [33, 35, 37, 45]; *Parasabanema* Smales & Heinrich, 2010 with one species [45]; *Pogonomystrongylus* Smales, 2014 with one species [36]; *Nugininema* Smales, 2016 and *Rodentanema* Smales, 2016 with one species each [38]; *Parvinema* Smales, 2017 with one species [40]; *Missimstrongylus* Smales, 2018 with one species [41]; *Flannerystrongylus* Smales, 2019 with two species [42, 43]; *Helgenema* Smales, 2020 with two species [43, 44]; and *Paramelomystrongylus* Smales, 2020 with one species [43].

**Table 1:** Genera of Nippostrongylinae – and their species – erected between 2009 and 2021 from New Guinean rodents (chronological order). Abbreviations: NG, New Guinea; PNG, Papua New Guinea.

Genus	Species	Host(s)	Distribution	Taxa concerned/Status
*Melomystrongylus* Smales, 2009	*Melomystrongylus sepikensis* Smales, 2009	*Melomys rufescens*	PNG	Valid
		*Melomys* spp.		
	*Melomystrongylus somoroensis*	*Paramelomys rubex*	NG	Valid
	Smales & Heinrich, 2010			
*Mawsonema* Smales & Heinrich, 2010	*Mawsonema mokwanense* Smales & Heinrich, 2010	*Pa. rubex*	NG	*M. mokwanense sp. inq*.
				Nippostrongylinae *i.s.* 1
				*Mawsonema gen. inq*.
*Montistrongylus* Smales & Heinrich, 2010	*Montistrongylus ingati* Smales & Heinrich, 2010	*Pa. rubex*	PNG	*M. ingati sp. inq.* Nippostrongylinae *i.s*. 2
	*Montistrongylus giluwensis* Smales, 2011	*Coccymys ruemmleri*	PNG	*M. giluwensis sp. inq.*
	*Montistrongylus karungi* Smales, 2012	*Abeomelomys sevia*	PNG	*M. karungi sp. inq.*
				Nippostrongylinae *i.s.* 3
	*Montistrongylus kaindiensis* Smales, 2015	*Pogonomys sylvestris*	PNG	*M. kaindiensis sp. inq.*
				*Montistrongylus gen. inq*.
*Parasabanema* Smales & Heinrich, 2010	*Parasabanema szalayi* Smales & Heinrich, 2010	*Pa. rubex*	NG	Valid
	*Parasabanema sene* Smales, 2020	*Paramelomys mollis*	PNG	Valid
*Pogonomystrongylus* Smales, 2014	*Pogonomystrongylus domaensis* Smales, 2014	*Pogonomys loriae*	PNG	Valid
*Nugininema* Smales, 2016	*Nugininema titokis* Smales, 2016	*Rattus niobe*	PNG	Valid
*Rodentanema* Smales, 2016	*Rodentanema aenigma* Smales, 2016	*R. niobe*	PNG	*R. aenigma sp. inq*.
				Herpetostrongylinae *i.s.*
				*Rodentanema gen. inq.*
*Parvinema* Smales, 2017	*Parvinema bafunminense* Smales, 2017	*Paramelomys lorentzii*	PNG	*P. bafunminense sp. inq.*
		*Mammelomys lanosus*		Nippostrongylinae *i.s.* 4
	*Parvinema helgeni* Smales, 2017	*Mammelomys rattoides*	PNG	*P. helgeni sp. inq.*
		*P. lorentzii*		*Parvinema gen. inq.*
*Missimstrongylus* Smales, 2018	*Missimstrongylus oweni* Smales, 2018	*Rattus verecundus*	PNG	*M. oweni sp. inq. Missimstrongylus gen. inq.*
*Flannerystrongylus* Smales, 2019	*Flannerystrongylus abulus* Smales, 2019	*Paramelomys platyops*	NG	*F. abulus sp. inq.*
	*Flannerystrongylus chisholmae* Smales, 2020	*Paramelomys levipes*	NG	*F. chisholmae sp. inq.*
		*Pa. mollis*		*Flannerystrongylus gen. inq.*
*Helgenema* Smales, 2020	*Helgenema keablei* Smales, 2020	*Pa. levipes*	PNG	*H. keablei sp. inq.*
				*Nippostrongylinae i.s.* 5
	*Helgenema lamia* Smales, 2021	*Chiruromys lamia*	PNG	*H. lamia sp. inq.*
		*Chiruromys forbesi*		*Helgenema gen. inq.*
*Paramelomystrongylus* Smales, 2020	*Paramelomystrongylus dessetae* Smales, 2020	*Pa. mollis*	NG	*P. dessetae sp. inq.*
				*Nippostrongylinae i.s.* 6
				*Paramelomystrongylus gen. inq*.

In addition to the above-mentioned genera and species, another 26 species were described, distributed into the following genera (Table 2):

*Bunomystrongylus* Hasegawa & Mangali, 1996 (one species) [37].
*Hasanuddinia* Hasegawa & Syafruddin, 1994 (three species) [32, 36, 37].
*Hasegawanema* Durette-Desset & Digiani, 2015 (one species) [42].
*Heligmonoides* Baylis, 1918 (one species) [30].
*Hughjonestrongylus* Digiani & Durette-Desset, 2014 (eight species) [29, 30, 32, 40, 42, 43, 45].
*Macrostrongylus* Ow-Yang *et al.,* 1983 (one species) [29].
*Odilia* Durette-Desset, 1973 (eight species) [29–31, 36, 37, 39].
*Paraheligmonelloides* Fukumoto, Kamiya & Suzuki, 1980 (three species) [40, 45].

**Table 2:** Species of Nippostrongylinae described between 2009 and 2021 from New Guinean rodents (alphabetical order). *described as *Paraheligmonelloides*. **described as *Heligmonoides*. ***described as *Odilia*. Abbreviations: PI, Papua Indonesia; PNG, Papua New Guinea.

Genus	Species	Host(s)	Distribution	Comments
*Bunomystrongylus* Hasegawa & Mangali, 1996	*Bunomystrongylus ilami* Smales, 2015	*Pogonomys championi*	PNG	Nippostrongylinae *i.s*. related to *Bunomystrongylus*
*Equilophos* Durette-Desset & Digiani, 2015	*Equilophos similis* (Smales, 2009)***	*Melomys rufescens*	PNG	Valid after Durette-Desset & Digiani (2015)
*Hasanuddinia* Hasegawa & Syafruddin, 1994	*Hasanuddinia chiruromyos* Smales, 2011	*Chiruromys vates*	PNG	Valid
	*Hasanuddinia hasegawai* Smales, 2015	*Pogonomys sylvestris*	PNG	Valid
	*Hasanuddinia pogonomyos* Smales, 2014	*Pogonomys macrourus*	PNG	Valid
		*Pogonomys loriae*		
*Hasegawanema* Durette-Desset & Digiani, 2015	*Hasegawanema yuroense* Smales, 2019	*Paramelomys platyops*	PNG	Nippostrongylinae *i.s*. (careen absent)
*Hughjonestrongylus* Digiani & Durette-Desset, 2014	*Hughjonestrongylus alisoni* Smales, 2020	*Paramelomys mollis*	PI	Valid
	*Hughjonestrongylus amplicaudae* (Smales & Heinrich, 2010)*	*Paramelomys rubex*	PI	Valid after Digiani & Durette-Desset (2014)
	*Hughjonestrongylus arfakiensis* Smales, 2020	*Pa. mollis*	PI	Valid
	*Hughjonestrongylus digianiae* Smales, 2020	*Pa. mollis*	PNG	Valid
	*Hughjonestrongylus ennisae* (Smales & Heinrich, 2010)*	*Pa. rubex*	NG	Valid after Digiani & Durette-Desset (2014)
	*Hughjonestrongylus implexus* (Smales, 2008)***	*Uromys caudimaculatus Uromys anak*	PNG	Valid after Digiani & Durette-Desset (2014)
	*Hughjonestrongylus mirzai* (Smales, 2009)**	*Melomys rufescens*	PNG	Valid after Digiani & Durette-Desset (2014)
	*Hughjonestrongylus pervulgatus* Smales, 2019	*Pa. platyops*	NG	Valid
		*Melomys* sp.		
	*Hughjonestrongylus singauwaensis* (Smales & Heinrich, 2010)*	*M. rufescens*	PNG	Valid after Digiani & Durette-Desset (2014)
		*Pa. rubex*		
	*Hughjonestrongylus spratti* Smales, 2020	*Pa. mollis*	PNG	Valid
	*Hughjonestrongylus vanimoensis* Smales, 2019	*Pa. platyops*	PNG	Valid
	*Hughjonestrongylus wanumaensis* Smales, 2019	*Pa. platyops*	PNG	Valid
	*Hughjonestrongylus wooleyae* Smales, 2017	*Paramelomys lorentzii*	PNG	Valid
			Aru Island	
	*Hughjonestrongylus* sp*.* of Smales (2011)*	*C. vates*	PNG	Valid after Digiani & Durette-Desset (2014)
*Lesleyella* Durette-Desset & Digiani, 2015	*Lesleyella wauensis* (Smales, 2010)***	*Lorentzimys nouhuysi*	PNG	Valid after Durette-Desset & Digiani (2015)
*Macrostrongylus* Ow-Yang, Durette-Desset & Ohbayashi, 1983	*Macrostrongylus ingens* Smales, 2008	*U. caudimaculatus*	NG	*Macrostrongylus* transferred in the Nippostrongylinae by Durette-Desset *et al*. (2017). *M. ingens* clearly related or similar to the type species *Macrostrongylus ratti*
		*M. rufescens*		
		*Melomys* sp*.*		
		*Paramelomys levipes*		
		*Pa. platyops*		
		*Pa. cf. platyops*		
*Odilia* Durette-Desset, 1973	*Odilia carinatae* Smales, 2008***	*U. anak*	PNG	Nippostrongylinae *i.s.* after Durette-Desset & Digiani (2015)
		*U. caudimaculatus*		
	*Odilia hagemannae* Smales, 2016	*Rattus giluwensis*	PNG	Nippostrongylinae *i.s.* related to *Maxomystrongylus*
	*Odilia helgeni* Smales, 2015	*Po. sylvestris*	PNG	Nippostrongylinae *i.s.* related to *Sanduanensis*
	*Odilia whittingtoni* Smales, 2015	*Po. sylvestris*	PNG	Nippostrongylinae *i.s.* related to *Sanduanensis*
*Sanduanensis* Durette-Desset & Digiani, 2015	*Sanduanensis dividua* (Smales, 2014)***	*Po. macrourus*	PNG	Valid after Durette-Desset & Digiani (2015)

In the course of the same period, and as part of a critical revision of the Nippostrongylinae from the Australasian region, Digiani & Durette-Desset (2014) [2] then Durette-Desset & Digiani (2015) [8] treated the systematic position of the species belonging to the genera *Paraheligmonelloides* and *Odilia*, respectively. Such revisions included 10 species from New Guinea described by Smales [29–32, 36] and Smales & Heinrich [45] in these two genera (Table 2). In 2014 [2], the genus *Paraheligmonelloides* was split into four genera, among which only one, *Hughjonestrongylus*, is present in New Guinea. In 2015 [8], several species of *Odilia* present in New Guinea were treated: five out of them were distributed into different genera, among which *Parasabanema*, that was considered valid (Table 2). Two other species, *Odilia uromyos* (Mawson, 1961) and *O. carinatae* Smales, 2008 were considered Nippostrongylinae *i.s*.

As part of a critical revision of the Nippostrongylinae, it seems to us important to address the validity of the genera and species described between 2008 and 2021, not treated in 2014 [2] or 2015 [8]. The geographical distribution of all taxa treated is also addressed.

## 2 Materials and methods

The genera (and the species they include) are treated herein in the chronological order of their erection (and/or description). Only the nominal species are treated. The data were compiled from the published descriptions. For each of the genera revised, we have carefully examined the original description and the illustrations of the type species, then of other species described in the genus. This procedure has often evinced difficulties of which the most frequent were as follows:

- For some parasitic species found in more than one host species, it was not specified if the description was only from the material from the type host.
- The measurements of the holotype were mixed with those of the paratypes. This procedure avoids the individualization of the holotype and consequently of the species, in the case of coparasitism by more than one taxon.

The morphological criteria used in the identification of a trichostrongyle rely essentially on characters of the synlophe and, to a lesser degree, of the bursa. The methods used for the study and description of the synlophe in the present work follow the terms and criteria provided by Durette-Desset [5], Durette-Desset & Digiani [6] and Durette-Desset & Digiani [8]. The methods used for the study and description of the bursa follow Durette-Desset & Digiani [7]. Concerning the synlophe, it is useful to make two points. First, the synlophe is generally similar in both sexes of the same species, the main difference being in the number of ridges, which is usually higher in the female because of its (generally) larger diameter. The similarity of the synlophe in both sexes allows the precise matching of males and females of the same species when dealing with coparasitism by several taxa. The second point is that in the Nippostrongylinae, the ridges are oriented either from the right-ventral to the left-dorsal quadrant or, perpendicularly to the body surface. In the first case, an axis of orientation of the ridges, oblique to subfrontal, can be recognized. In the second case, on the contrary, it is not possible to identify a preferential axis of orientation of the ridges.

Concerning the original synlophe descriptions of the genera treated, we often found the following difficulties:

- For a given species, the male synlophe was very different from that of the female.
- The level at which the sections was made was imprecise. This made the comparisons between related species or between both sexes of the same species difficult.
- The author indicated having studied the synlophes of several males and females. However, the sections illustrating different levels of the body were not indicated as coming from the same specimen or from different specimens.
- The lateral cords were not illustrated, or were misplaced. This hindered differentiating the dorsal/ventral surfaces and calculating the inclination of the axis of orientation of the ridges.
- The ridges were illustrated pointing in disparate directions. This hindered the interpretation of the orientation of the section.
- The absence of an axis of orientation of the ridges (when ridges are perpendicular to the body surface) was often confused with the existence of a subfrontal axis.
- The axis of orientation was not represented with an arrow but as a straight line (without arrowheads) or as a double arrow. Whereas, by definition, the axis of orientation of the ridges has one and only one direction.

Concerning the bursa, it was rarely fully expanded. In most cases, the drawings of the three lobes (left, right and dorsal) were provided separately. The orientation of the lobes was rarely specified.

Finally, there is often a contradiction between the text and the illustrations.

It is often claimed, in the original descriptions of the taxa mentioned above, that in spite of the fragility of the material studied, the morphological data were sufficient to characterize the new genera. We do not completely agree with this view; regardless of the condition of the material, very often descriptions are incomplete or ambiguous and the illustrations show several inconsistencies and are difficult to interpret. Consequently, after careful examination of the descriptions and the illustrations we sought to interpret the latter by relying on the elements already known in the Nippostrongylinae in order to, secondarily, amend the descriptions. This procedure, in several cases, allowed us to clarify the status of a given genus, but it was not always possible.

Within the present work, the numbering of the figures appears frequently double (two elements separated by a slash). In such cases, the first number refers to the figure numbering in the original published description of the taxon; the second one, to our own numbering. The latter is always indicated by a number and letter. The number identifies the plate (one per genus), whereas the letter identifies a given section or figure. A letter (*e.g.*, 1A) indicates the original drawing, whereas a letter followed by an apostrophe (*e.g.*, 1G’) means that the figure was reinterpreted (consequently modified) in the present work.

All body sections are oriented with the dorsal side of the worm towards the top of the page, and the left side of the worm towards the left of the page. All scale bars are 50 μm.

## 3 Results

Genera of Heligmonellidae described from New Guinean murids between 2009 and 2020.

### 3.1 Genus *MELOMYSTRONGYLUS* Smales, 2009 (Fig. 1)

**Type species:** *Melomystrongylus sepikensis* Smales, 2009.

**Figure 1:**
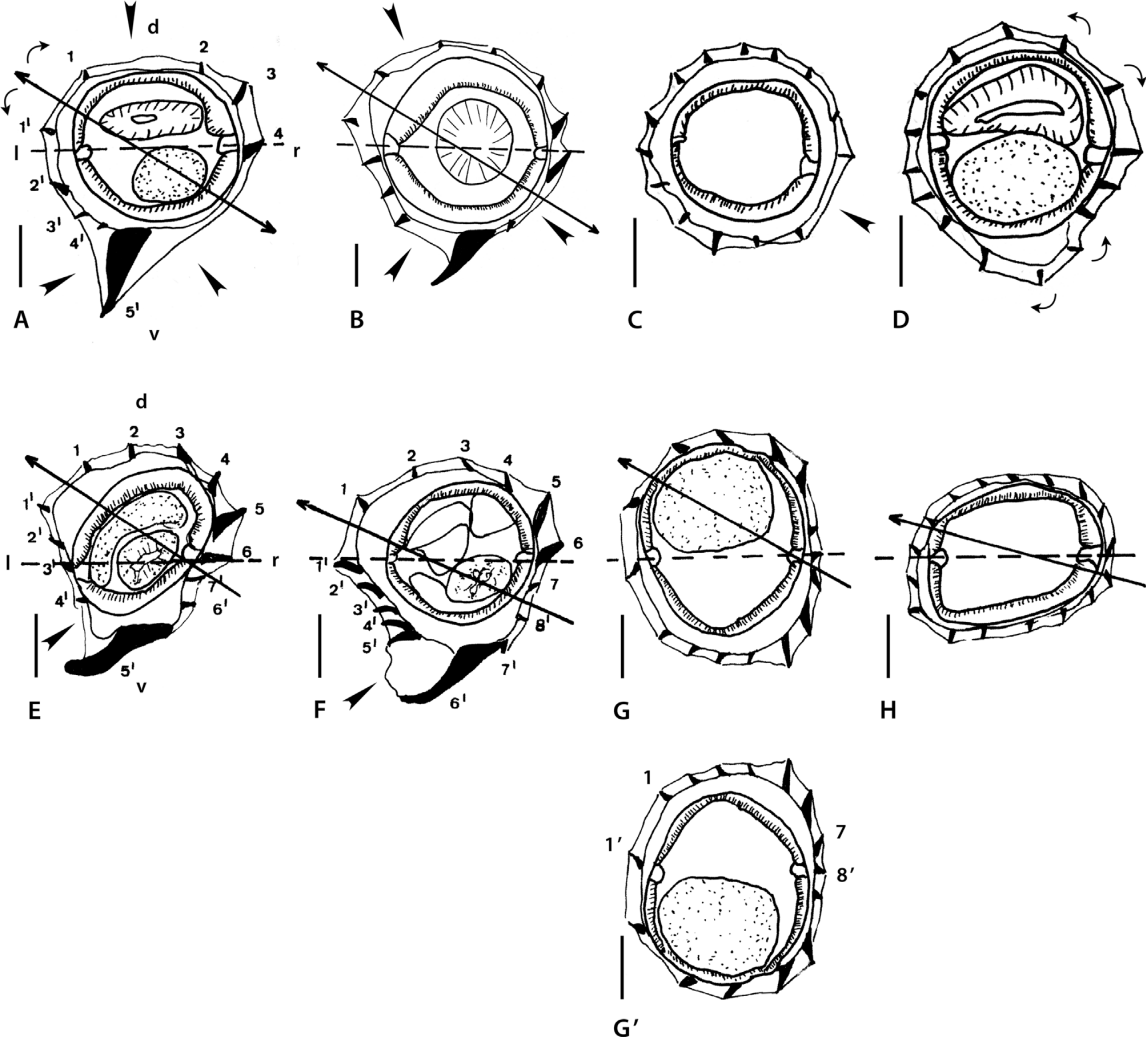
Genus *Melomystrongylus* Smales, 2009. Body sections. A–D *Melomystrongylus sepikensis* Smales, 2009. A, B within proximal body. A male, B female; C, D at midbody. C male, D, female. E–H *Melomystrongylus somoroensis* Smales & Heinrich, 2010. E, F within proximal body. E male, F female. G, G’ at midbody, male. H female “at posterior end of midbody” (sic). Sources: A–D redrawn from [30]. E–H redrawn from [45]. G’ modified figure, reversed on its frontal axis with respect to the original.

**Hosts:** Muridae, Hydromyinae (Rodentia).

**Site:** small intestine.

**Distribution:** Papua New Guinea.

**Other species:** *Melomystrongylus somoroensis* Smales & Heinrich, 2010.

**Original diagnosis:** *Trichostrongyloidea: Heligmonellidae: Nippostrongylinae. Synlophe well developed with pointed ridges; in midbody axis of orientation of ridges passing through ventral right and dorsal left sides inclined about 65° from sagittal axis in anterior body, lacking clear orientation in mid and hind body. Ventral ridge 5’ hypertrophied anteriorly. Bursa asymmetrical with larger left lobe. Dorsal ray divided distal to level of branching of rays 8 from dorsal trunk. Parasites of hydromyine murids* [30].

#### 3.1.1 Analysis of data and difficulties encountered

##### 3.1.1.1 *Melomystrongylus sepikensis* (Figs. 1A–1D)

###### 3.1.1.1.1 Synlophe (number of worms studied not provided)

Sections analyzed herein are those within proximal part of body: male (Fig. 16/1A) and female (Fig. 17/1B), and at midbody: male (Fig. 19/1C) and female (Fig. 20/1D). In all sections, lateral cords illustrated; ridges numbered in Figure 16/1A.

**Figure 2:**
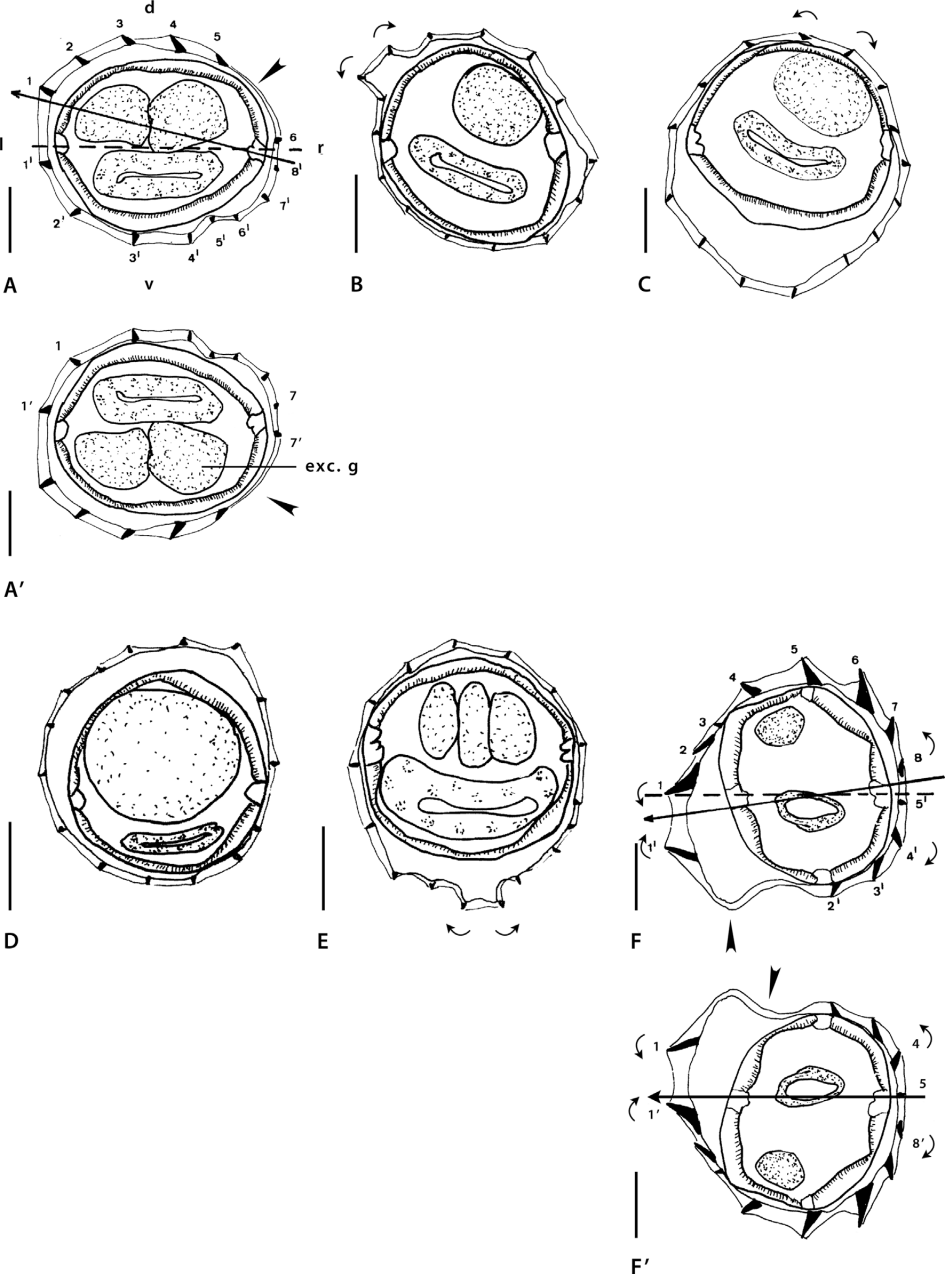
Genus *Mawsonema* Smales & Heinrich, 2010. Body sections. A–F’ *Mawsonema mokwanense* Smales & Heinrich, 2010. A, A’ within proximal body, male. B, C at midbody. B male, C female. D, E within distal body. D male, E female. F, F’ within proximal body, female. A–E synlophe of type I. F, F’ synlophe of type II. Source: A–F redrawn from [45]. A’, F’ modified figures: reversed on their frontal axes with respect to the originals. F’ axis of orientation reinterpreted as subfrontal.

*Within proximal body*: in both sexes, careen absent, synlophe with hypertrophied ventral ridge (ridge 5’, interpreted herein as comarete) and two large right ridges associated to right lateral cord; axis of orientation described as oblique by Smales [30].

Figure 1A (male section): nine ridges irregularly spaced with three gaps (arrowheads) of which largest situated on right-ventral quadrant; tips of ridges 1 and 1’ divergent (curved arrows).

Figure 1B (female section): 12 ridges less irregularly spaced; gaps much smaller than those in male section (arrowheads).

*At midbody:* Figures 1C and 1D, synlophe very different from that within proximal body; ridges increased in number (14–16), small and subequal in size, lacking clear orientation. All features characterizing synlophe at proximal part disappear.

Figure 1C (male section): all ridges subequal with no systematic orientation, most oriented perpendicularly to body surface; small ridge-free space present on right-ventral quadrant (arrowhead).

Figure 1D (female section): right ventral ridges largest; two pairs of ridges with divergent tips present (curved arrows): one right-dorsal and one right-ventral; no ridges with convergent tips observable, remaining ridges mostly perpendicular to body surface.

###### 3.1.1.1.2 Bursa (illustrated in [30]: Figs. 22 and 25)

Figure 22: dorsal ray and rays 8, orientation not specified. Figure 25: left lobe, left ray 8 and left branch of dorsal ray, lateral view.

From the written description [30]: bursa dissymmetrical with left lobe larger; left rays 2 and 3 longer than rays 4-6.

##### 3.1.1.2 *Melomystrongylus somoroensis* (Figs. 1E–1H)

###### 3.1.1.2.1 Synlophe (based on sections from five worms, sex not specified)

Sections analyzed herein are those within proximal part of body: male (Fig. 38/1E) and female (Fig. 40/1F), and at midbody: male (Fig. 42/1G), and female (Fig. 44/1H). In all sections lateral cords illustrated; ridges numbered in Figures 38/1E and 40/1F.

*Within proximal body*: in both sexes careen absent, synlophe with hypertrophied ventral ridge (ridge 5’, interpreted herein as comarete), and three large right ridges associated with right lateral cord; in both sections tips of ridges 1 and 1’ divergent; axis of orientation oblique.

Figure 1E (male section): 13 ridges almost regularly spaced: ridges on left-dorsal quadrant more widely spaced than those on right-dorsal quadrant. Gap between ridges 5’ and 4’ (arrowhead).

Fig. 1F (female section): 15 ridges irregularly spaced with left ventral ridges tighter than other ridges. Gap between ridges 6’ and 5’ (arrowhead).

*At midbody:* in both sexes careen absent, synlophe differing markedly from that in proximal part (comarete disappeared, number of ridges slightly increased). Ridges losing orientation after Smales & Heinrich [45].

Figure 1G (male section): ridges unequal in size and oriented from right to left on both dorsal and ventral sides, except tip of left ridge directed ventrally and convergent with tip of ridge immediately ventral.

Figure 1H (female section): ridges rather subequal in size, regularly spaced and oriented from right-ventral to left-dorsal side.

###### 3.1.1.2.2 Bursa (illustrated in [45]: Fig. 49)

Figure 49: bursa flattened out, orientation not specified; rays 2 and 3 longer than rays 4-6. From the written description [45]: bursa dissymmetrical with right lobe larger; pattern of type 3-2.

#### 3.1.2 Comments

##### 3.1.2.1 Synlophe

*Size of ridges*: the “midbody” sections of male and female seem to have been taken at different levels. Specifically, the female sections were probably taken towards the end of the mid-region of body (*i.e.*, more distally than the male sections). This would explain the differences concerning the ridge size, since ridges usually become subequal towards the posterior end. This can be confirmed in the type species by noticing the male section at distal body (Fig. 46 in [45]) which is identical to the “midbody” female section (Fig. 1H). Unfortunately, in neither of the two species are there intermediate sections between the proximal body and the midbody. Consequently, it is not possible to know at which levels the ridges start decreasing in size, or new ridges appear. This means that it is not possible to establish any homologies between the synlophe at midbody and that within the proximal part of body.

*Orientation of ridges:* for both species, it was indicated [30, 45] that the orientation of the ridges is oblique within the proximal body and unclear in the rest of the body. Concerning the proximal part, we agree with the original statement, though this implies that on the four illustrated proximal sections, the tips of the ridges 1 and 1’ (which are supposed to be convergent) are incorrectly figured.

At midbody, the orientation of the ridges is unclear in the type species (Figs. 1C and 1D) but it is more evident in the female section of *M. somoroensis* where the ridges determine an oblique, almost subfrontal, axis of orientation (Fig. 1H). In the male section of *M. somoroensis* (Fig. 1G), the fact that the left ridge is oriented toward the ventral side means that the pair of ridges with convergent tips (curved arrows on the left) is situated ventrally. Such arrangement actually determines an axis of orientation right-dorsal to left-ventral, which is not at all the rule in the Heligmonellidae. We propose the reversion of the section on its frontal axis, which results in the left ridge directed dorsally and an axis of orientation directed from right-ventral to left-dorsal quadrant (Fig. 1G’). Although without a clear inclination, this arrangement is similar to that observed in the female section (Fig. 1H).

As noted by Smales [30], *Melomystrongylus* is characterized by the absence of a careen and the presence of a hypertrophied mid-ventral ridge within the proximal third of the body. Among the Australasian Nippostrongylinae, there is only one genus without a careen and with hypertrophied ventral ridges: *Hasanuddinia*. Smales [30] distinguished *Melomystrongylus* from *Hasanuddinia* by the presence of a single hypertrophied ridge in the first third of body length, *versus* three hypertrophied ridges along the whole body in *Hasanuddinia*.

##### 3.1.2.2 Bursa

*M. sepikensis:* judging from the illustration, the pattern of the left lobe is 1-4 with ray 3 diverging at same level as ray 6 from their common trunk. The pattern of the right lobe is not illustrated.

*M. somoroensis:* judging from the illustration, the pattern is 1-3-1 for both lobes with rays 3 and 6 diverging at same level from their common trunk.

In the Remarks concerning *M. somoroensis*, Smales & Heinrich [45] noted:

“*The generic diagnosis of the genus* Melomystrongylus *includes “bursa asymmetrical, left lobe larger”, the determination having been made after examining bursae that had not been completely rolled flat. In* M. somoroensis *the right lobe is the larger one and since this could also be the case for* M. sepikensis *the diagnosis needs to be reconsidered.*”

#### 3.1.3 Conclusion

Despite the incomplete description of the synlophe, the presence of a hypertrophied ventral ridge (interpreted herein as a comarete) within the proximal third of the body allows the characterization of the genus. We consider *Melomystrongylus* a valid genus. Further studies will be needed, especially of the bursa of the type species, to provide a more complete generic definition.

#### 3.1.4 Emended diagnosis

*Melomystrongylus.* Synlophe without careen. Within anterior third of body, 9–12 ridges; presence of one ventral comarete; ridges irregularly spaced; right ridge and dorsal adjacent ridge largest; other ridges unequal in size and small. At mid-body, 14–17 ridges medium-sized to small, irregularly spaced. Axis of orientation oblique within proximal part, uncertain in midbody. Characteristic bursal pattern of type 1-3-1 (only known in *M. somoroensis*).

### 3.2 Genus *MAWSONEMA* Smales & Heinrich, 2010 (Fig. 2)

**Type and sole species:** *Mawsonema mokwanense* Smales & Heinrich, 2010.

**Hosts:** Muridae, Murinae, Hydromyini (Rodentia).

**Host site:** small intestine.

**Distribution:** New Guinea.

**Original diagnosis:** *Nippostrongylinae. Synlophe well developed, with 15 continuous longitudinal pointed ridges: anterior body with axis of orientation of ridges sub frontal in anterior, lacking orientation in mid and hind body. Bursa asymmetric, left lobe largest. Pattern of bursal rays 2-3. Dorsal ray divided distal to level of branching of rays 8 from dorsal trunk. Parasites of hydromyine murids* [45].

#### 3.2.1 Analysis of data and difficulties encountered *Mawsonema mokwanense*

##### 3.2.1.1 Synlophe (based on sections from 10 worms, sex not specified)

Sections analyzed herein are within proximal body: male (Fig. 21/2A) and female (Fig. 23/2F), at midbody: male (Fig. 24/2B) and female (Fig. 27/2C), and within distal body: male (Fig. 28/2D) and female (Fig. 33/2E). Lateral cords illustrated in all figures and ridges numbered in Figures 21/2A and 23/2F. Axis of orientation described as subfrontal in [45] within proximal part, lacking orientation in middle and distal part.

*Within proximal body (male)*: in Figure 2A, careen absent; 15 ridges small to minute, regularly spaced; presence of gap on right-dorsal quadrant (arrowhead). Most ventral ridges perpendicular to body surface; dorsal ridges oriented from right to left. Excretory glands observed in dorsal position.

*At midbody (both sexes)*: in Figures 2B and 2C, careen absent; 15 minute ridges regularly spaced. In Figure 2B (male), on left-dorsal quadrant, small dilatation present supported by two ridges whose tips are slightly divergent (curved arrows). Remaining ridges perpendicular to body surface. Figure 2C (female), on dorsal, right-dorsal quadrant, presence of 2 minute ridges pointing to opposite directions (curved arrows); remaining ridges oriented mainly from right-dorsal to ventral side.

*Within distal body (both sexes)*: in Figures 2D and 2E, careen absent; in Figure 2D (male) 14 ridges, in Figure 2E (female) 16 ridges minute and regularly spaced. Most ridges apparently perpendicular to body surface although in female, presence of small, mid-ventral dilatation supported by seven ridges including two with tips apparently divergent (curved arrows).

*Within proximal body (female)*: in Figure 2F, careen present (curved arrows on the left), made up of two ridges of which dorsal one larger than ventral one; 13 ridges (including careen) medium-sized to minute, almost regularly spaced, except for large gap on left-ventral quadrant (arrowhead). Tips of ridges 8 and 4’ divergent (curved arrows on the right); tip of ridge 5’ perpendicular to body surface. Remaining ridges oriented from right to left on dorsal and ventral sides. Axis of orientation of ridges oriented few degrees above frontal axis on right side, below frontal axis on left side.

##### 3.2.1.2 Bursa (based on 12 worms, illustrated in [45]: Figs. 32, 34 and 37)

Figure 32: entire bursa partially unfolded, orientation not specified. Figure 34: distal part of rays 8 and dorsal ray, orientation not specified; rays not illustrated up to base. Figure 37: entire bursa closed showing ventral rays.

From the written description [45]: bursa dissymmetrical with left lobe larger than right one and pattern of type 2-3 in both lobes.

#### 3.2.2 Comments

##### 3.2.2.1 Synlophe

Within the proximal part of the body, in the male section (Fig. 2A) the dorsal position of the excretory glands seems at first sight unlikely since these glands reach the excretory pore, which is ventral by definition. However, the position of the excretory glands may be dorsal if the level of the section is distant enough from the excretory pore. Based only on the position of these glands, and without information on the exact level of the section, we cannot know if the dorso-ventral orientation of the section is right or not. Nevertheless, if Fig. 2A is reversed on its frontal axis (Fig. 2A’), the gap becomes right-right-ventral, as it is frequent in the Nippostrongylinae (arrowhead). Despite this reversion, there are still no groups of ridges oriented in opposite directions, and consequently it is not possible to determine an axis of orientation of the ridges.

At midbody and within distal part of body (Figs. 2B–2E), except for the presence of a pair of clearly divergent ridges in Figure 2C, the orientation of the ridges is rather disparate and it is likely that most ridges are in fact oriented perpendicularly to the body surface. The reason for the absence of an axis of orientation in these sections is the perpendicular orientation of the ridges, not a “loss of orientation” of these latter (as interpreted in [47].

Within the proximal part of another female (Fig. 2F), several elements allow us to suggest that the original orientation of the section is erroneous: (1) the axis of orientation of the ridges is oriented from right dorsal side from left ventral side; (2) the dorsal ridge of the careen is larger than the ventral one; (3) the largest ridges are dorsal in position; and (4) dorsal ridges are more numerous than the ventral ones.

Usually, in the Nippostrongylinae, (1) the axis of orientation of the ridges is oriented from the right-ventral to the left-dorsal quadrant or, at most, subfrontal; (2) when the ridges of the careen are unequal in size, the ventral ridge (1’) is always the largest; (3) other developed ridges are in mid-ventral or left-ventral position (never mid-dorsal); and (4) the ventral ridges are usually more (or as) numerous than the dorsal ridges.

For the orientation to be accurate, Figure 2F should be reversed on its frontal axis (Fig. 2F’). In the re-oriented section, the axis of orientation of the ridges becomes subfrontal, it is determined by ridges 4 and 8’ (divergent) on the right (ridge 5 being perpendicular to body surface) and 1 and 1’ (convergent) on the left (curved arrows).

Smales & Heinrich [45] stated in the “Remarks”: “*Mawsonema* n. gen. has all the characteristics of the subfamily Nippostrongylinae except that the orientation of synlophe ridges exceeds the range given by Durette-Desset [4]”. We believe that the “range” to which the authors refer is the range of the inclination of the axis of orientation of the ridges which, following [4], in the Nippostrongylinae ranges between 45° and 67° to the sagittal axis. However, since 1983, the separation of subfamilies based only on the inclination of the axis of orientation has become less reliable since, based on more recent data, the inclination of the axis in the Nippostrongylinae actually ranges from 25° to 90° to the sagittal axis, a range which overlaps with that of the Pudicinae and the Brevistiatinae [1].

Nevertheless, we interpret the “exceptional” inclination (below the frontal axis) alleged by Smales & Heinrich [45] in *Mawsonema* as a misinterpretation of the orientation of the section 2F (treated above).

##### 3.2.2.2 Bursa

In Figure 32, judging from the bursal shape and the position of the drawing strokes, the bursa is in ventral view. The ray bases are not illustrated. In Figure 37, only the divergence of rays 2 and 3 is illustrated.

Without description or illustration of the origin or level of divergence of rays 4-6 and 8, the bursal pattern cannot be confirmed.

#### 3.2.3 Conclusion

##### 3.2.3.1 Synlophe

Smales & Heinrich [45] studied the synlophe on 10 specimens but only 6 body sections were illustrated. It is not indicated if all body sections provided were taken from six different worms or made at different levels on one male and one female. Anyway, it is possible to state that the female synlophe illustrated on Figure 2F is very different from the others by having a well-developed careen, leading us to suggest that two different genera are present within the studied material. Based on the elements listed above, two types of synlophe could be described:

*Type I*: characterized by the absence of careen along whole body. With 15 ridges in male, 17 in female at mid-body, 15 in male, 16 in female within distal part. Ridges subequal, minute and regularly spaced. Within proximal part, ventral and left-ventral ridges oriented from right to left. At midbody and within distal part of body, no axis of orientation, most ridges being apparently perpendicular to body surface (Figs. 2A–2E).

*Type II*: Only represented by a female section within proximal part of body (Fig. 2F). Characterized by a careen supported by two small ridges. With 13 ridges including the careen. Gap present on left-dorsal quadrant. Ridges of right-ventral quadrant most developed, but not larger than the ridges of the careen. Axis of orientation probably subfrontal.

##### 3.2.3.2 Bursa

From the illustrations and the written description, it is not possible to confirm the pattern of the bursa. Twelve worms were studied but only one is described and illustrated, whereas two types of synlophe have been highlighted. We have no data to attribute the described bursa to a given type of synlophe.

There seem to be two taxa concerned in the description of this species (each characterized by a different synlophe): “*M. mockwanense*” and a Nippostrongylinae *i.s.* 1. Since we do not know what type of synlophe the holotype corresponds to, *Mawsonema mokwanense* is considered a *species inquirenda*. Being the type species of the genus, it is impossible to give a precise definition of it. We thus consider *Mawsonema* a *genus inquirendum*.

### 3.3 Genus *MONTISTRONGYLUS* Smales & Heinrich, 2010 (Fig. 3)

**Type species:** *Montistrongylus ingati* Smales & Heinrich, 2010.

**Figure 3a:**
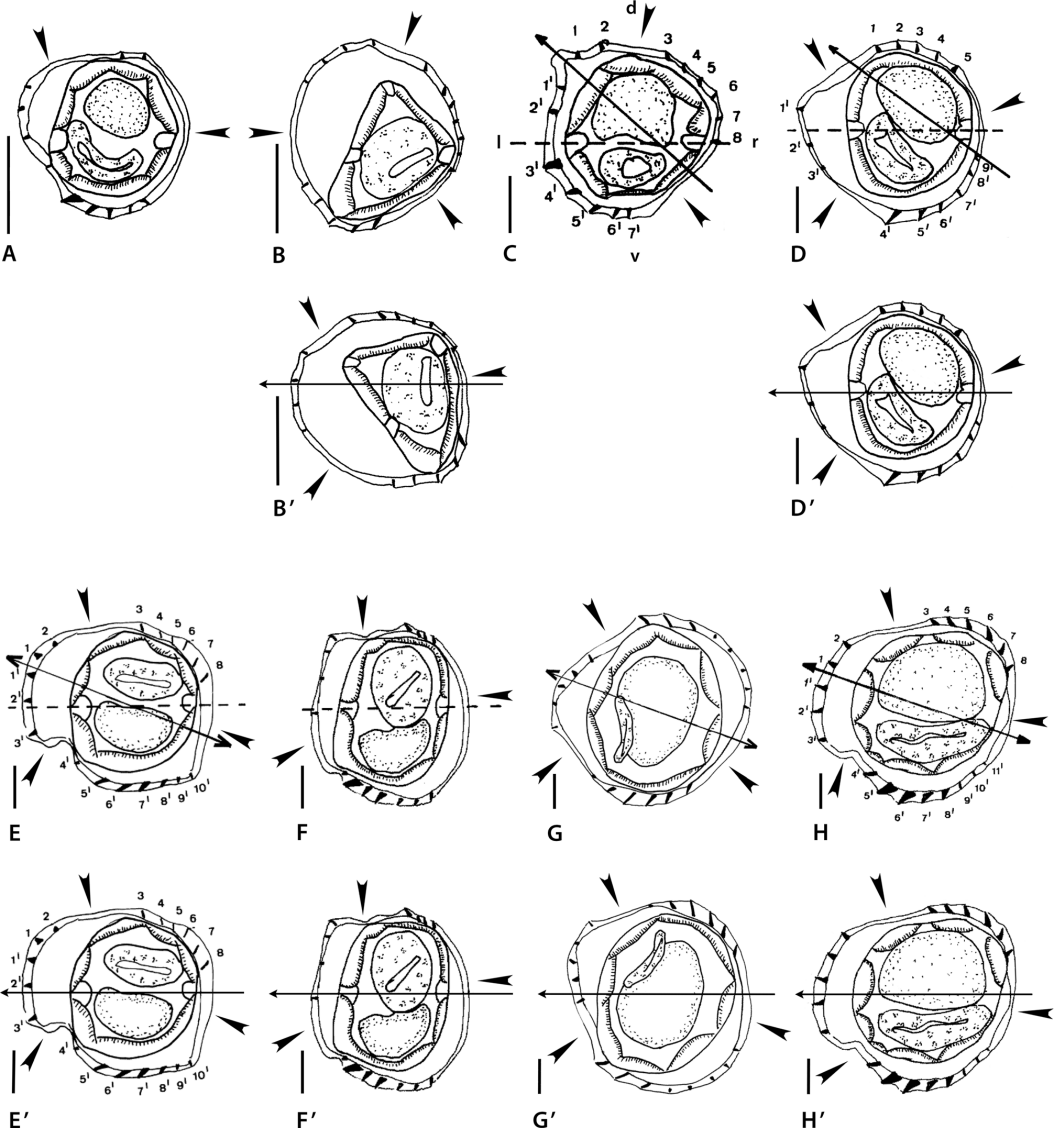
*Montistrongylus* Smales & Heinrich, 2010. Body sections. A–D’ *Montistrongylus ingati* Smales & Heinrich, 2010. A–B’ within proximal body. A male, B, B’ female. C–D’ at midbody. C male, D, D’ female. E–H’ *Montistrongylus giluwensis* Smales, 2011. E, F within proximal body. E male, F female. G–H’ at midbody. G, G’ male, H, H’ female. Sources: A, D redrawn from [45]; E–H redrawn from [33]. B’, D’, G’, H’ modified figures: B’ rotated *ca*. 75° counterclockwise with respect of the original. D’–H’ reinterpreted orientation of the axis (subfrontal). G’ reversed on its frontal axis then rotated 30° clockwise with respect to the original. H’ rotated *ca*. 10–20° counterclockwise with respect to the original.

**Hosts:** Muridae, Murinae (Rodentia).

**Host site:** small intestine.

**Distribution:** Papua New Guinea.

**Other species:** *Montistrongylus giluwensis* Smales, 2011; *Montistrongylus karungi* Smales, 2012; *Montistrongylus kaindiensis* Smales, 2015.

**Original diagnosis:** *Nippostrongylinae. Synlophe with up to 15 small pointed ridges, left ventral ridges largest: axis of orientation of ridges from ventral right to dorsal left, 55° from sagittal axis at mid body, lacking careen. Bursa slightly asymmetrical, right lobe larger, dorsal lobe about same length as lateral lobes. Pattern of bursal rays 3-2. Rays 8 asymmetrical, left ray longer. Dorsal ray divided distal to level of branching of rays 8 from dorsal trunk. Parasites of hydromyine murids* [45].

#### 3.3.1 Analysis of data and difficulties encountered

In the four species described, a careen is absent and the ridges are grouped into two or three sets alternating with two or three ridge-free spaces. Herein, for the description of the synlophe of each species, the ridge sets will be numbered (set 1, set 2, set 3) clockwise starting from the left (set 1). Axis of orientation described as oblique all along body by Smales & Heinrich [45].

##### 3.3.1.1 *Montistrongylus ingati* (Figs. 3A–3D')

###### 3.3.1.1.1 Synlophe (based on sections from six worms, sex not specified)

Sections analyzed herein are those within proximal body: male (Fig. 51/3A) and female (Fig. 52/3B) and at midbody: male (Fig. 54/3C) and female (Fig. 55/3D). Lateral cords illustrated; ridges numbered in sections at midbody.

*Within proximal body:* in Fig. 3A (male), two ridge sets alternating with two ridge free-spaces (arrowheads); ridge set 1 on left and ventral sides made up of nine ridges: four left ridges similar in size, perpendicular to body, smaller and more spaced than five ventral ridges with decreasing gradient in size from left to right, oriented from right to left except last ridge on the right; ridge set 2 made up of two minute ridges perpendicular to body.

Figure 3B (female): three ridge sets alternating with three ridge free-spaces (arrowheads); ridge set 1 on left-dorsal side, made up of four minute ridges perpendicular to body; ridge set 2 on right-dorsal quadrant made up of seven minute ridges without clear orientation; ridge set 3 on mid-ventral side made up of five minute ridges, oriented from right to left.

*At midbody:* in Figure 3C (male), two ridge sets alternating with two ridge-free spaces (arrowheads); ridge set 1 on left side, made up of nine small ridges (7’-1’, 1 and 2) similar in size, except ridges ventrally adjacent to left lateral cord (3’ and 4’), slightly larger; ridges regularly spaced; ridges 3’-1’, 1 and 2 plus ridge 7’ perpendicular to body surface; other ventral ridges slightly oriented from right to left. Ridge set 2 on right- dorsal quadrant, made up of six small ridges (3 to 8) similar in size and oriented from right to left (ridges 3 to 6) or perpendicularly to body surface (ridges 7 and 8).

Figure 3D (female), three ridge sets alternating with three ridge-free spaces (arrowheads); ridge set 1 in front of left lateral cord, made up of three small ridges (3’ to 1’) similar in size and perpendicular to body surface; ridge set 2 on mid-dorsal quadrant, made up of five small ridges (1 to 5) similar in size, oriented from right to left except ridge 5, perpendicular to body surface; ridge set 3 on right-ventral quadrant, made up of six ridges (9’ to 4’) of which 4’ and 5’ slightly larger, all oriented from right to left.

###### 3.3.1.1.2 Bursa (based on 7 worms; illustrated in [45]: Figs. 63 and 65)

Figure 63: dorsal ray and rays 8, orientation not specified; rays 8 dissymmetrical. Figure 65: bursa flattened out, orientation not specified, symmetrical diverging of rays 8 at base of dorsal ray. From the original written description [45]: bursa slightly dissymmetrical with right lobe larger; dissymmetrical divergence of rays 8 from median part of dorsal ray; pattern of type 3-2 in both lobes.

##### 3.3.1.2 *Montistrongylus giluwensis* (Figs. 3E–3H’)

###### 3.3.1.2.1 Synlophe (based on sections from six worms, sex not specified)

Sections analyzed herein are those within proximal part of body: male (Fig. 4/3E) and female (Fig. 14/3F) and at midbody: male (Fig. 9/3G) and female (Fig. 16/3H); lateral cords illustrated in Figures 4/3E and 14/3F; ridges numbered in Figures 4/3E and 16/3H; careen absent.

In all sections, three ridge sets alternating with three ridge-free spaces (arrowheads).

*Within proximal body:* in Figure 3E (male), ridge set 1 situated on left side in front of left lateral field, made up of five small ridges (3’-1, 1 and 2) similar in size; regularly spaced and oriented perpendicularly to body surface; ridge set 2 situated on right-dorsal quadrant, made up of six small ridges (3 to 8) similar in size, regularly spaced and oriented from right to left; ridge set 3 situated on ventral side, made up of seven small ridges, ridges 5’ to 7’ slightly larger than other ridges; ridges regularly spaced with ridges 5’ to 7’ oriented from right to left, other ridges oriented perpendicularly to body surface.

Figure 3F (female): ridge set 1 situated on left-dorsal quadrant, made up of four small ridges similar in size, regularly spaced (with large spaces) and oriented perpendicularly to body surface; ridge set 2 situated on dorsal, right-dorsal side, made up of four small ridges, similar in size, regularly spaced (with small spaces) and oriented from right to left; ridge set 3 situated on mid-ventral side, made up of seven ridges of which the left ones slightly larger (except the last ridge one, minute), all ridges oriented from right to left.

*At midbody:* in Figure 3G (male), ridge set 1 situated on left side, made up of five small ridges unequal in size, regularly spaced and oriented from right to left (three dorsal ridges) or perpendicularly to body surface (other two ridges); ridge set 2 situated on right-dorsal side, made up of eight small ridges oriented from right to left (three dorsal ridges) or perpendicularly to body surface (other ridges); ridge set 3 situated on mid-ventral side, made up of six small ridges, median three being slightly larger, oriented from right to left.

Figure 3H (female): ridge set 1 situated on left side, made up of five ridges (3’ to 1’, 1 and 2), almost similar in size, regularly spaced, oriented perpendicularly to body surface; ridge set 2 situated on right-dorsal side, made up of six small ridges, almost similar in size, regularly spaced and oriented from right to left; ridge set 3 situated on ventral side, made up of eight small ridges (5’, 6’ largest, then 4’, 7’, 8’ then 9’ to 11’ minute) regularly spaced and oriented from right to left except ridges 9’ to 11’ oriented perpendicularly to body surface.

###### 3.3.1.2.2 Bursa (number of worms studied not specified, illustrated in [33]: Figs. 10, 12 and 13)

Figure 10: distal part of dorsal ray with rays 8, dorsal view; right ray 8 clearly extending beyond division of dorsal ray. Figure 12: left lobe and left proximal part of dorsal ray, dorsal view. Figure 13: right lobe and right proximal part of dorsal ray, dorsal view; right ray 8 just reaching level of division of dorsal ray. From the original written description [33]: bursa symmetrical with right lobe slightly larger than left one and pattern of type 2-3 in both lobes.

##### 3.3.1.3 *Montistrongylus karungi* (Figs. 3I–3K)

###### 3.3.1.3.1 Synlophe (based on sections from four worms, sex not specified)

Sections analyzed herein are those within proximal part of body: male (Fig. 2/3I), and at midbody: male (Fig. 4/3J) and female (Fig. 12/3K). In all sections lateral cords illustrated and ridges numbered in Figures 4/3 J and 12/3K.

**Figure 3b:**
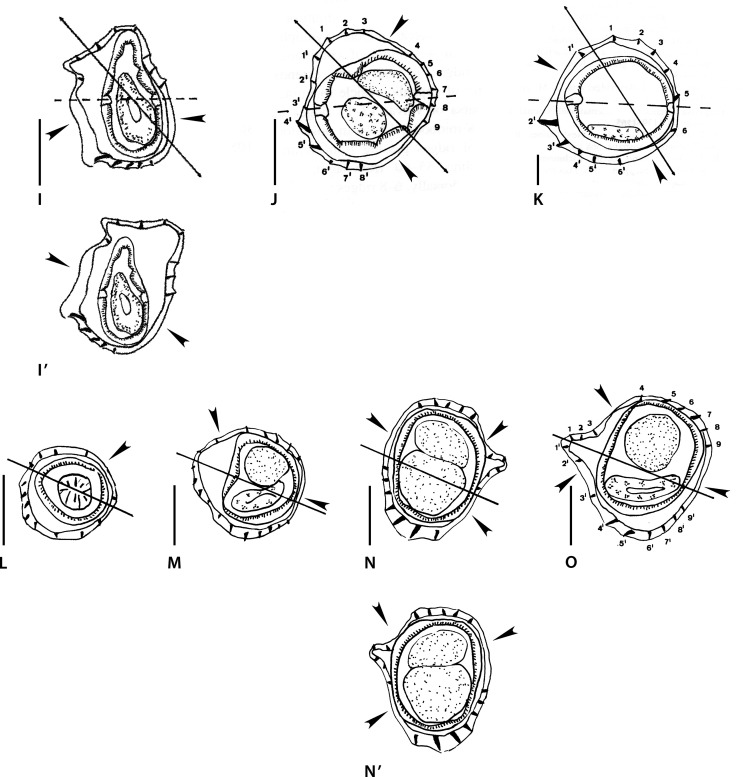
I–K *Montistrongylus karungi* Smales, 2012. I, I’ within proximal body, male. J, K at midbody. J male, K female. L–O *Montistrongylus kaindiensis* Smales, 2015. L, M within proximal body. L male, M female. N–O at midbody. N, N’ male, O female. Sources: I–K redrawn from [35]; L–O redrawn from [37]. I’, N’, modified figures. I’ body displaced inside the cuticle to obtain a section congruent with Figure 3K. N’ reversed on its sagittal axis then rotated 30° clockwise with respect to the original.

In all sections ridges irregularly spaced and grouped into two sets (set 1, set 2) alternating with two ridge-free spaces (arrowheads).

*Within proximal body:* in Figure 3I (male), 13 ridges; ridge set 1 situated on dorsal side, made up of eight small ridges, similar in size, irregularly spaced and oriented perpendicularly to body surface; ridge set 2 situated on mid-ventral side, made up of five small ridges regularly spaced with slight decreasing gradient in size from left to right and oriented from right to left.

*At midbody:* in Figure 3J (male), 17 ridges; set 1 situated on left side, made up of 11 small ridges (8’ to 1’ and 1 to 3) similar in size, except ridges ventrally adjacent to left lateral cord (4’ and 5’), slightly larger; most ridges oriented perpendicularly to body surface; only ridges 5’ and 4’ oriented from right to left; ridge set 2 situated on mid-right side, made up of six small ridges (4 to 9), similar in size, regularly spaced, without clear orientation.

Figure 3K (female): 11 ridges; ridge set 1 situated on left-ventral quadrant, made up of five small ridges regularly spaced, ridges ventrally adjacent to lateral cord (3’, 2’) larger and oriented from right to left, other ridges (6’-4’) smaller and oriented perpendicularly to body surface; ridge set 2 situated mainly on right-dorsal quadrant, extending to both mid-dorsal and mid-right sides, made up of seven small ridges (1’ and 1 to 6); ridges 1’, 5 and 6 apparently oriented from right-ventral to left dorsal-quadrant, remaining ridges (1 to 4) apparently perpendicular to body surface.

###### 3.3.1.3.2 Bursa (number of worms studied not specified; illustrated in [35]: Figs. 7 and 11)

Figure 7: dorsal ray and rays 8, dorsal view. Figure 11: bursa spread out, dorsal view. From the original written description [35]: bursa slightly dissymmetrical with right lobe larger and pattern of type 2-3 in both lobes.

##### 3.3.1.4 *Montistrongylus kaindiensis* (Figs. 3L–3O)

###### 3.3.1.4.1 Synlophe (number of worms studied not specified)

Sections analyzed herein are within proximal body: male (Fig. 28/3L), female (Fig. 27/3M) and at midbody: male (Fig. 30/3N) and female (Fig. 31/3O); lateral cords not illustrated; ridges numbered in Figure 31/3O.

*Within proximal body*: Figure 3L (male), judging from drawing, is made at level of esophagus; 13 small ridges irregularly spaced; large gap on right-dorsal quadrant (arrowhead); left ridges oriented from dorsal to ventral side; orientation of other ridges uncertain.

Figure 3M (female): judging from drawing, section made at level of intestine; 18 small ridges irregularly spaced; ridges separated into two groups by two large gaps (arrowheads).

*At midbody:* in both sections, ridges grouped into three sets alternating with three ridge-free spaces (arrowheads). Position of ridge sets and that of ridge-free spaces differing between both sexes.

Figure 3N (male): ridge set 1 situated on left-ventral quadrant, made up of seven small to medium sized ridges (not numbered) of which two ventral ones more developed and spaced, ridges oriented from ventral to left; ridge set 2 situated on mid-dorsal side, made up of five small ridges similar in size, oriented from dorsal to left (2 ridges) and from dorsal to right (3 ridges); ridge set 3 situated on mid-right side, made up of five minute ridges similar in size and oriented apparently perpendicularly to body surface.

Figure 3O(female): ridge set 1 situated on left side, made up of five minute ridges (2’, 1’ and 1 to 3) regularly spaced and oriented perpendicularly to body surface; ridge set 2 situated on dorsal side, made up of six small ridges, almost similar in size and regularly spaced except ridge 4; ridge set 3 situated on ventral side, made up of seven small ridges, irregularly spaced with ridges 4’ and 5’ larger. Ridges of sets 2 and 3 oriented in same direction: from left to right on dorsal side and from right to left on ventral side.

###### 3.3.1.4.2 Bursa (number of worms studied not specified; illustrated in [37]: Figs. 37 and 38)

Figure 37: right lobe and dorsal lobe, orientation not specified. Figure 38: left lobe, orientation not specified; left ray 8 not illustrated. From the original written description [37]: bursa slightly dissymmetrical with right lobe larger and pattern of type 2-3.

#### 3.3.2 Comments

##### 3.3.2.1 Montistrongylus ingati

###### 3.3.2.1.1 Synlophe

The two male sections (Figs. 3A, 3C) differ from each other and seem not to belong to the same taxon. In addition, they have two major differences with the female sections (Figs. 3B, 3D): there are two sets of ridges in male *versus* three in female and, on the other hand, in the female sections, the axis of orientation is subfrontal (Fig. 3D’) and not oblique, as illustrated in the original figures. In the female proximal body, to be congruent with the female section at midbody, the section should be rotated *ca.* 75° counterclockwise (Fig. 3B’).

###### 3.3.2.1.2 Bursa

From the original written description, the left ray 8 is longer than right ray 8 and the right lobe is slightly larger than left one. This means that Figures 63 and 65 are in dorsal view. The pattern is 2-2-1 in the right lobe and 2-3 in the left lobe. The illustration of two types of dorsal lobes (Figs. 63 and 65) reinforces the idea that two types of males are present among the type material, *i.e.*, probably two different taxa. We have no information to attribute the described bursae to a given type of synlophe.

##### 3.3.2.2 Montistrongylus giluwensis

###### 3.3.2.2.1 Synlophe

The indication of the lateral cords in both sexes within the proximal part of the body and the position of the ridge sets and ridge free spaces (Figs. 3E and 3F) allows us to re-orientate the sections at midbody. The reversion of the male section on the frontal axis then a slight rotation clockwise (Fig. 3G’) results in both sections (male and female) having the same pattern of ridge sets and ridge-free spaces, but also the same number of ridges in the respective ridge sets, with dorsal ridges less numerous than ventral ones. In Figure 3H, a slight rotation counterclockwise results in an axis of orientation subfrontal (Fig. 3H’).

###### 3.3.2.2.2 Bursa

Unlike the original written description, the illustration of both latero-ventral lobes, highlights the right lobe being clearly larger that the left one. One explanation could be that the lobes illustrated do not belong to the same species. This hypothesis is reinforced by the illustration of two types of dorsal lobe. The first type (Fig. 10) is illustrated independently of the latero-ventral lobes; it is characterized by a right ray 8 extending beyond the level of the division of the dorsal ray. It could be linked to the left lobe (Fig. 12) but this remains hypothetical. The second type (Fig. 13) is illustrated with the right lobe. Even if it is not completely illustrated, it is characterized by a right ray 8 just reaching the level of the division of the dorsal ray. This means that, among the males studied, at least two taxa are present. From the original illustration, the pattern is of type 1-4 in both lobes with a short common trunk of rays 3-6; in the right lobe (Fig. 13) rays 4 to 6 diverge at the same level from their common trunk, in the left lobe, rays 6 diverge proximally to rays 4 and 5.

##### 3.3.2.3 Montistrongylus karungi

###### 3.3.2.3.1 Synlophe

The descriptions of the three sections of *M. karungi* (Figs. 3I, 3J, 3K) are similar with those of the male sections of *M. ingati* (Figs. 3A, 3C): two sets of ridges alternating with two ridge-free spaces. The orientation of the axis remains uncertain, many ridges being oriented perpendicularly to body surface. However, the position of the ridge sets and ridge-free spaces differ between the three sections.

The proximal section of a male (Fig. 3I) has been deformed during fixation, and the body itself, limited by the hypodermis is not at its correct place. If we displace the body inside the cuticle (Fig. 3I’), we obtain a section in which the position of the ridges and the ridge sets is very similar with the section of the female at midbody. In addition, the number of ridges in the two sections is similar (11 *vs.* 13) as opposed to 17 for the section of a male at midbody.

This latter section is closely related to the section of a male of *M. ingati* at midbody: same position of the sets, 15 ridges in *M. ingati*, 17 in *M. karungi.*

###### 3.3.2.3.2 Bursa

From the illustration, the bursa is of type 1-4 in both lobes; rays 3 diverging from a common trunk at the same level as ray 6 in the right lobe and proximally to it in the left lobe. Right ray 6 diverges first from the common trunk 4-6 in right lobe, at about same level in the left lobe. There are clearly two different types of dorsal lobes which means that there are probably two different taxa among the males studied.

##### 3.3.2.4 Montistrongylus kaindiensis

###### 3.3.2.4.1 Synlophe

Concerning the male section of the proximal body (Fig. 3L), the presence of only one ridge-free space and then the absence of ridge sets prevents us from relating this synlophe to any of the remaining sections studied. A section of the esophagus is observed, which means that the body section has been taken very proximally. Thus, it is difficult to compare the male with the female “proximal” body section (Fig. 3M), taken much more distally. The latter, in addition, has two ridge sets *vs*. three in the midbody sections.

The female section at midbody (Fig. 3O) seems congruent with the other *Montistrongylus* spp. by ridge set 1 situated on the left. Instead, the orientation of the male section (Fig. 3N) is less clear. The reversion of the male section on its sagittal axis and further rotation about 30° clockwise (Fig. 3N’) results in both sections at midbody (male and female) having a similar pattern of ridge sets and ridge-free spaces. Such manipulation of the male section changes the original ridge set 3 into set 1 and the ridge set 1 into set 3.

In spite of the clear presence of three sets of ridges alternating with three ridge-free spaces, this synlophe description shows several inconsistencies. Apart from the ridges of set 1, which are perpendicular to body surface, the remaining ridges show an anarchical orientation. Ridges in set 2 in the male section (regardless of the orientation of the section) are oriented in opposite directions, so that there are divergent ridges *within* the ridge set. By contrast, in the female section, all ridges of set 2 are oriented from left to right, contrarily to what is usual for the dorsal ridges in the Nippostrongylinae. Ridges of set 3 in the reoriented male section (Fig. 3N’) are oriented from left to right, contrarily to what is expected for the ventral ridges in the Nippostrongylinae; whereas in the female section, the same ridges are oriented, as expected, from right to left (Fig. 3O). The disparate orientation of most ridges prevents the identification of an axis of orientation and does not allow a reliable interpretation of the synlophe. Moreover, no attempt at reorientation of any of these sections will make them comparable to each other or to any other species in the genus or in the family. We are not able to interpret these particularities or to state whether both sections correspond to the same species.

###### 3.3.2.4.2 Bursa

From the original illustration, the pattern is of type 1-4 in both lobes with rays 3 diverging proximally to rays 6, and rays 6 diverging first from common trunk to rays 4-6.

#### 3.3.3 Conclusion

##### 3.3.3.1 Synlophe

From a descriptive point of view we observed, throughout the descriptions, three types of synlophe.

Type I is observed in the male sections at midbody of *M. ingati* and *M. karungi* (Figs. 3C, 3J): it is characterized by two sets of ridges alternating with two ridge-free spaces; and an oblique axis of orientation of the ridges.

Type II is observed in the female section at midbody of *M. ingati* (Fig. 3D’) and the male and female sections of *M. giluwensis* (Figs. 3G’, 3H’): it is characterized by three sets of ridges alternating with three ridge-free spaces; and an axis of orientation subfrontal at midbody. It would be possible to relate to this second type the male and female sections of *M. kaindiensis* (Figs. 3N’, 3O) based on the presence of three ridge sets and three ridge-free spaces, but the orientation of the ridges in these two sections is completely disparate.

Type III is observed in a male section within the proximal part of the body (Fig. 3I’) and in a female section at midbody in *M. karungi* (Fig. 3K): it is characterized by two sets of ridges alternating with two ridge-free spaces. This type differs from the male section of *M. karungi* at midbody (Fig. 3J) by the position of the dorsal ridge-free space situated on the left side in the female *versus* the right side in the male.

That said, we can state that, besides the absence of a careen, all the taxa described within *Montistrongylus* may be characterized by the alternation of 2 or 3 sets of ridges with 2 or 3 ridge-free spaces and ridges small and more or less regularly spaced within the sets.

##### 3.3.3.2 Bursa

Even if the original descriptions of *M. ingati, M. giluwensis* and *M. karungi* describe only one type of bursa, the illustrations show two types of dorsal lobes in each species mentioned, which probably means that there are at least two different taxa within each “species” studied. For *M. kaindiensis*, the illustration of the dorsal lobe is incomplete, the right ray 8 not being figured.

Though the taxa described within *Montistrongylus* are characterized by ridge sets alternating with ridge-free spaces, and ridges small and regularly spaced within the sets, *Montistrongylus* is considered a *genus inquirendum*. In our interpretation, the type species is composed of two different taxa, each represented by a body section with different synlophe types: type I in the male and type II in the female. In the article by Smales & Heinrich [45], a male was designated as holotype but its description is mixed with that of the paratypes and it is not possible to know if its synlophe corresponds to type I or to type II.

Since we do not know what type of synlophe the holotype corresponds to, *Montistrongylus ingati* is considered a *species inquirenda*. *Montistrongylus giluwensis* and *M. karungi* are also considered *species inquirendae* since their descriptions seem to involve two different taxa each. *Montistrongylus kaindiensis* is similarly considered a *species inquirenda* because its synlophe description shows several inconsistencies, which were explained above. The three types of synlophe recognized (type I, II and III) seem to be distributed into six different taxa as follows: “*M. ingati*” (male); “*M. karungi*” (male) (both with synlophe of type I); Nippostrongylinae *i.s.* 2 (“*M. ingati*” female); “*M. giluwensis”*; “*M. kaindiensis”* (all with synlophe of type II) and Nippostrongylinae *i.s.* 3 (“*M. karungi*” male section within proximal part of body and female section at midbody) (with synlophe of type III).

### 3.4 Genus *POGONOMYSTRONGYLUS* Smales, 2014 (Fig. 4)

**Type and sole species:** *Pogonomystrongylus domaensis* Smales, 2014.

**Figure 4:**
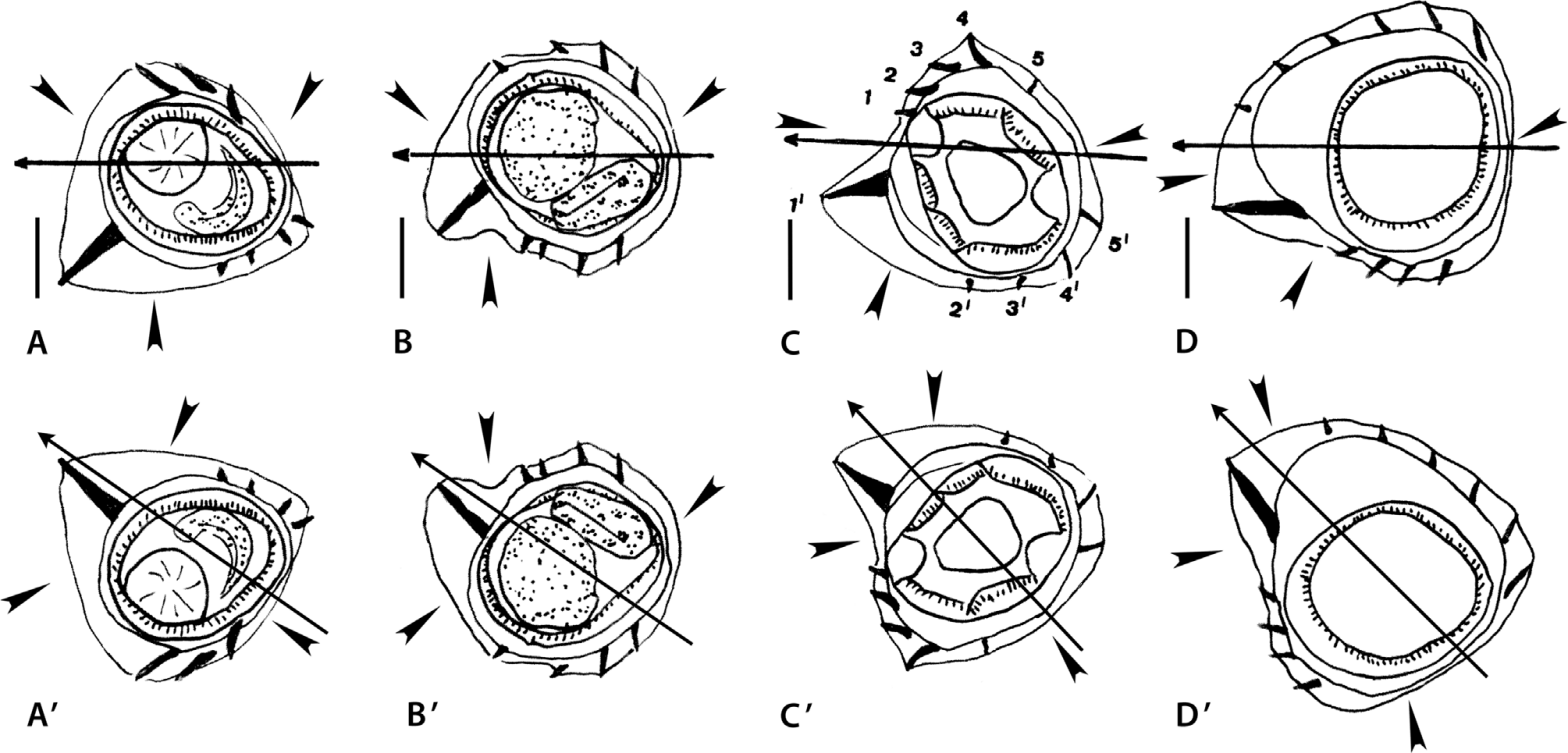
Genus *Pogonomystrongylus* Smales, 2014. Body sections. A–D’ *Pogonomystrongylus domaensis* Smales, 2014. A–B’ within proximal body. A, A’ male, B, B’ female. C–D’ at midbody. C, C’ male, D, D’ female. Source: A–D redrawn from [36]. A’–D’, modified figures: A’–C’ reversed on their frontal axes with respect to the originals, then slightly rotated clockwise. D’ rotated 45° clockwise.

**Hosts:** Muridae, Murinae (Rodentia).

**Host site:** small intestine.

**Distribution:** Papua New Guinea.

**Original diagnosis:** *Nippostrongylinae. Synlophe well developed with 7-10 continuous pointed longitudinal ridges, single left ventral ridge hypertrophied: axis of orientation of ridges sub frontal. Bursa asymmetrical, right lobe larger. Pattern of bursal rays 2-3, dorsal ray divided distal to branching of rays from dorsal trunk. Parasites of hydromyine murids* [36].

#### 3.4.1 Analysis of data and difficulties encountered

##### 3.4.1.1 Synlophe (number of worms studied not provided)

Sections analyzed herein are those within proximal body: male (Fig. 37/4A) and female (Fig. 38/4B), and at midbody: male (Fig. 40/4C) and female (Fig. 41/4D). Lateral cords illustrated and ridges numbered only in Figure 40/4C.

In all sections, careen absent and ridges grouped into three sets alternating with three ridge-free spaces, indicated by arrowheads. Ridge sets are numbered herein (as set 1, set 2, set 3) starting from left and clockwise. In all sections, position of the sets and ridge-free spaces the same: ridge set 1 situated on left side and made up of sole large ridge whose tip directed to ventral side (Figs. 4A, 4B, 4C) or perpendicular to body surface (Fig. 4D). Ridge set 3 made up of four small ridges regularly spaced and subequal, oriented from right to left. In all sections, axis of orientation described as subfrontal in [36].

*Within proximal body:* in Figure 4A (male), ridge set 2 made up of three medium-sized ridges regularly spaced oriented from right to left. Figure 4B (female), ridge set 2 made up of four ridges (two small, two medium-sized) oriented from right to left.

*At midbody:* in Figure 4C (male), ridge set 2 made up of five small ridges, ridges 4 to 1 showing decreasing size gradient and oriented from right to left plus minute ridge 5 perpendicular to body.

Figure 4D (female), ridge set 2 made up of six small to minute ridges oriented from right to left with decreasing size gradient from right to left.

##### 3.4.1.2 Bursa (number of worms studied not provided, illustrated in [36]: Figs. 42, 44 and 45)

Figure 42: dorsal ray and rays 8, orientation not specified. Figure 44: left lateral lobe and Figure 45: right lateral lobe. Genital cone developed (though “not prominent” from the written description). From the generic definition, bursa dissymmetrical with right lobe slightly larger and pattern of type 2-3.

#### 3.4.2 Comments

##### 3.4.2.1 Synlophe

In sections 4A and 4B, the ridges determine, in spite of the interpretation of Smales [36], an oblique axis of orientation directed from the right-dorsal to the left-ventral quadrant, and this is mainly due to the fact that the tip of ridge 1’ is directed to the ventral side. By definition, the axis of orientation of the Heligmosomoidea is directed from the right-ventral to the left-dorsal quadrant; in addition, in the Heligmonellidae, the tip of ridge 1’ is always directed to the dorsal side. If the sections are reversed on their frontal axis, then slightly rotated clockwise (Figs. 4A’, 4B’), the inclination of the axis of orientation becomes oblique (and not subfrontal), directed from the right-ventral to the left-dorsal quadrant.

In section 4C, the axis is not subfrontal since it does not pass through the lateral cords. If the section is reversed on its frontal axis, then slightly rotated clockwise, the tip of ridge 1’ is directed dorsally and the axis becomes oblique (Fig. 4C’).

In section 4D, it is sufficient to turn the section clockwise 45° to obtain a new interpretation (Fig. 4D’) which is congruent with the re-oriented sections 4A’–4C’.

##### 3.4.2.2 Bursa

Captions and drawings contradict each other. In Figure 44, the genital cone is illustrated above the rays, which means that the lobe is seen in ventral view, and it is actually the right lobe. In Figure 45, the dorsal lobe is clearly visible and at the right side of the figure is folded ventrally which means that the bursa is seen in dorsal view and the illustrated lobe is actually the left lobe. Figure 42 is clearly in dorsal view. The genital cone is clearly prominent since its length attains 50% of the bursal length. Judging from the illustrations, the pattern is 1-4 in both lobes. In right lobe, ray 2 very short, ray 3 diverging first from common trunk to rays 3-6, then ray 6. In left lobe, rays 2 and 3 very long, rays 3 and 6 diverging at same level from their common trunk.

#### 3.4.3 Conclusion

If our interpretation of the synlophe and bursa are accurate, we consider *Pogonomystrongylus* a valid genus. However, due to the ambiguous descriptions of both the synlophe and bursa, further studies either on the type material or on new material would be necessary to provide a new definition of the genus. *Pogonomystrongylus* shares with *Melomystrongylus*, *Hasanuddinia* and *Montistrongylus* Smales, 2010 the following features: (1) careen absent and (2) alternation of ridge sets with ridge-free spaces. *Pogonomystrongylus* is distinguished from the three mentioned genera by the absence of ventral comaretes and by the presence of a large left ridge.

#### 3.4.4 Emended diagnosis

*Pogonomystrongylus.* Synlophe without careen. Ten ridges in male, 11 in female. Ridges irregularly spaced, grouped into three sets alternating with three ridge-free spaces. Ridge 1’ largest, flanked on both sides by ridge-free spaces. Other ridges small to medium-sized. Slight decreasing gradient in ridge size from right to left on both dorsal and ventral sides. Axis of orientation oblique. Bursal characteristic pattern of type 1-4 in both lobes.

### 3.5 Genus *NUGININEMA* Smales, 2016 (Fig. 5)

**Type and sole species:** *Nugininema titokis* Smales, 2016.

**Figure 5:**
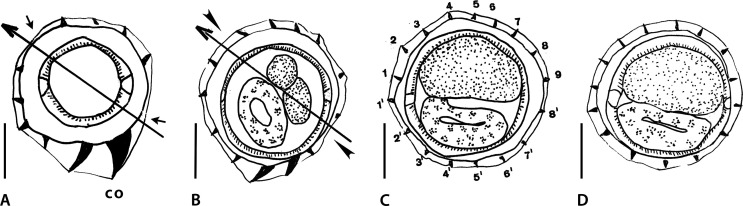
Genus *Nugininema* Smales, 2016. Body sections. A–D. *Nugininema titokis* Smales, 2016. A, B within proximal body. A male. B female. C, D, at midbody. C male, D female. Abbreviations: co, comarete. Source: A–D redrawn from [38].

**Hosts:** Muridae, Murinae (Rodentia).

**Host site:** small intestine.

**Distribution:** Papua New Guinea.

**Original diagnosis:** *Heligmonellidae, Nippostrongylinae. Synlophe well developed with 9-10 (anterior body) to 17 (midbody) continuous pointed longitudinal ridges, ventral ridge and adjacent right ventral ridge hypertrophied anteriorly; axis of orientation from right ventral to left dorsal side in anterior* (sic) *at about 50° to sagittal plane, axis subfrontal at mid body. Bursa dissymmetrical, right lobe larger, pattern of rays 1-3-1, dorsal ray divided distally to branching of rays 8. Parasites of murines, Rattini, from the island of New Guinea* [38].

#### 3.5.1 Analysis of data and difficulties encountered *Nugininema titokis*

##### 3.5.1.1 Synlophe (based on sections from two males and two females)

Sections analyzed herein are those within proximal body: male (Fig. 16/5A) and female (Fig. 17/5B), and at midbody: male (Fig. 19/5C) and female (Fig. 20/5D). In all figures, lateral cords illustrated except right lateral cord of section 19/5C; ridges numbered only in Fig. 19/5C.

In all sections careen absent. Axis of orientation described as oblique within proximal body and subfrontal at midbody by Smales [38].

*Within proximal body:* in Figure 5A (male), 10 ridges separated into two groups by two gaps, situated on left-dorsal quadrant and on right-ventral quadrant (arrowheads). On ventral side, presence of two large comaretes. Remaining ridges small and subequal in size. Most ridges oriented from right-ventral to left-dorsal quadrant, except: two left dorsal ridges flanking axis of orientation (perpendicular to body surface), and ventral ridge situated left to lesser comarete (oriented from left to right).

Figure 5B (female), 13 ridges of which two large ventral comaretes (in same position than in male section, but smaller). Remaining ridges small and subequal in size and irregularly spaced. Two gaps in the same position than in male section, but smaller (arrowheads). Dorsal ridges and comaretes oriented from right-ventral to left-dorsal quadrant. Remaining ventral ridges show disparate orientation, perpendicular or from left to right.

*At midbody:* in Figures 5C (male) and 5D (female), 15–17 ridges, regularly spaced, small, subequal and oriented disparately.

From the written description, axis of orientation “prefrontal” (sic) at midbody and losing orientation posteriorly.

##### 3.5.1.2 Bursa (number of studied males not specified; illustrated in [38]: Fig. 26)

Figure 26: right lobe, with right ray 8 and right branch of dorsal ray. From description [38], bursa dissymmetrical with right lobe slightly larger and pattern of type 1-3-1.

#### 3.5.2 Comments

##### 3.5.2.1 Synlophe

Within the proximal body (Figs. 5A and 5B) we interpret, as Smales [38], that the axis of orientation of the ridges is oblique. At midbody (Figs. 5C and 5D), instead, Smales [38] stated that the axis of orientation is “prefrontal” an affirmation that seems to us inaccurate. The disparate orientation of the ridges seems to be due to a misinterpretation, and it is likely that most ridges are oriented perpendicularly to the body surface. This means that there are not two groups of ridges with opposite directions, consequently, there is no axis of orientation of the ridges. It is clear that between the proximal body and the midbody, the number of ridges increased and the comaretes decreased progressively in size up to attain the same size as the other ridges. However, since the level of the “proximal” sections has not been specified and, besides, it is not equivalent in the male and the female, it is not possible to establish either homology of the male and female ridges or the level at which the ridges become subequal and perpendicular to the body surface.

##### 3.5.2.2 Bursa

Without illustration of the left lobe, and the right lobe being folded ventrally, it is difficult to determine the pattern of the rays or the symmetry of the bursa.

As noted by Smales [38] the genus *Nugininema* is related to the genera *Melomystrongylus* and *Hasanuddinia* by the presence of at least one ventral comarete and an axis of orientation of the ridges oblique within the proximal part of the body. In *Melomystrongylus* and *Nugininema,* the comaretes are present only in the proximal part of the body, whereas in *Hasanuddinia* they are present all along the body length. There are two comaretes in *Hasanuddinia* and *Nugininema* and only one in *Melomystrongylus*. *Nugininema* also differs from *Melomystrongylus* and *Hasanuddinia* by the fact that in the proximal body all dorsal ridges are small and subequal, whereas in the other two genera, the ridges associated with the right lateral cord (one or two ridges) are more developed.

#### 3.5.3 Conclusion

Concerning the synlophe, *Nugininema* can be differentiated unambiguously from the related genera. We thus consider *Nugininema* a valid genus. However, it would be necessary to describe and illustrate accurately the bursa to provide a proper definition of the genus.

#### 3.5.4 Emended diagnosis

*Nugininema*: Synlophe without careen. Within proximal part of body, presence of two ventral comaretes and two gaps: right-ventral and left-dorsal; remaining ridges small and subequal; axis of orientation of ridges oblique. At midbody, 15–17 ridges regularly spaced, small and subequal; ridges oriented perpendicularly to body surface.

### 3.6 *RODENTANEMA* Smales, 2016 (Fig. 6)

**Type species:** *Rodentanema aenigma* Smales, 2016.

**Figure 6:**
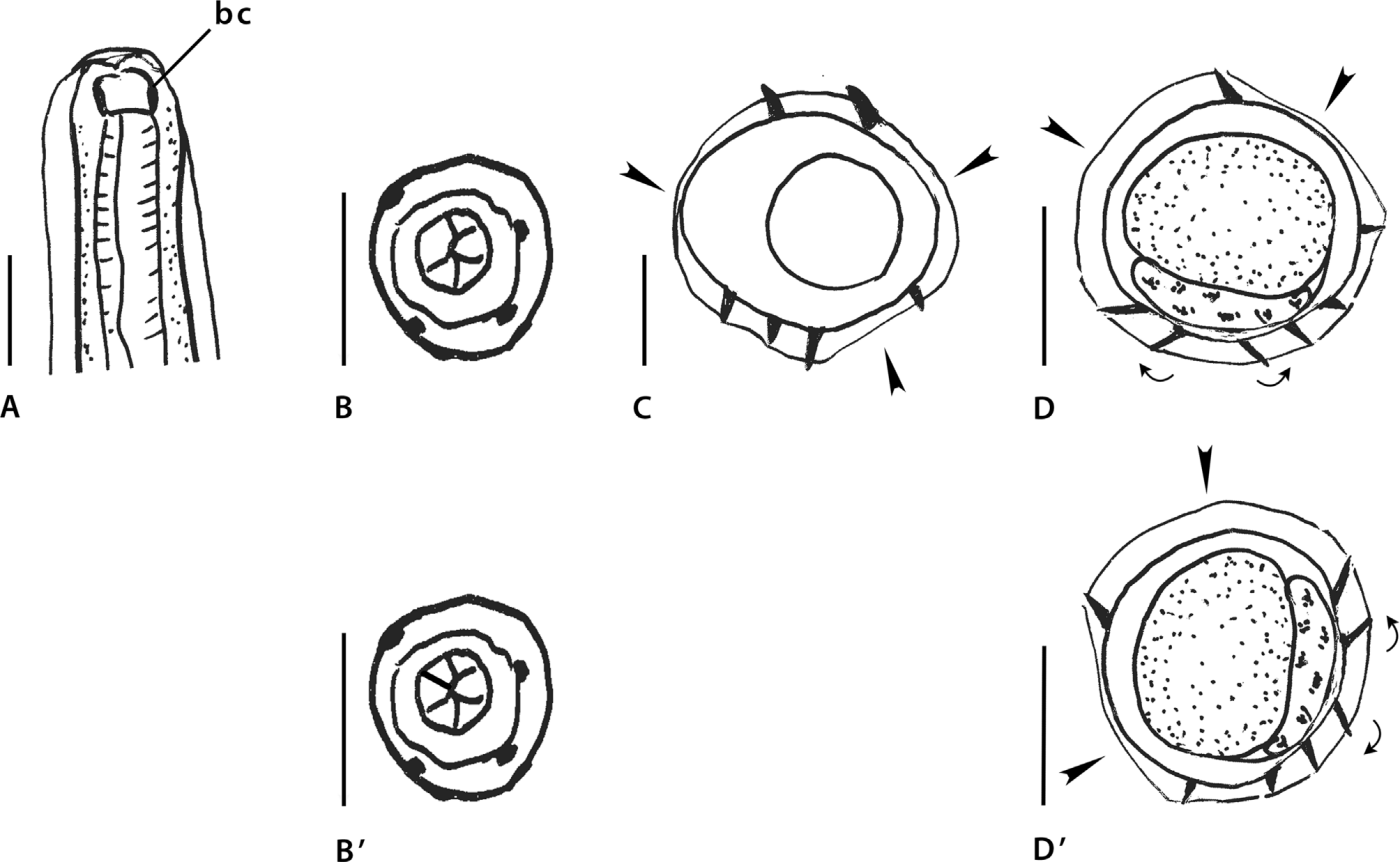
Genus *Rodentanema* Smales, 2016. A–D’. *Rodentanema aenigma* Smales, 2016. A–B’ head. A lateral or median view showing buccal capsule. B, B’ apical view. C, D sections at midbody. C male, D, D’ female. Abbreviations: bc buccal capsule. Source: A–D redrawn from [38]. B’, D’ modified figures: B’ six lips instead five. D’ rotated 90° counterclockwise with respect to the original.

**Hosts:** Muridae, Murinae (Rodentia).

**Host site:** small intestine.

**Distribution:** Papua New Guinea.

**Original diagnosis**: *Heligmonellidae. Nippostrongylinae. Synlophe well developed with 6-7 continuous pointed longitudinal ridges mid body. Buccal capsule relatively well developed, without lips or teeth. Cephalic vesicle present. Bursa dissymmetrical, right lobe larger, dorsal lobe short; pattern of lateral rays 1-4; dorsal ray divided close to distal end. Spicules simple, filiform. Female monodelphic; small number eggs in utero; tail without terminal spike. Parasites of murine rodents indigenous to the island of New Guinea* [38].

#### 3.6.1 Analysis of data and difficulties encountered *Rodentanema aenigma*

##### 3.6.1.1 Head

Illustrations analyzed herein are proximal extremity in lateral (Fig. 27/6A) and apical (28/6B) views. Buccal capsule (Fig. 27/6A) and five lips (Fig. 28/6B), illustrated.

##### 3.6.1.2 Synlophe (based on sections from two males and two females)

Sections analyzed herein are at midbody: male (Figs. 31/6C) and female (32/6D). Lateral cords not illustrated; ridges not numbered.

*At midbody:* in both sexes, careen absent and ridges subequal, medium-sized. Ridges irregularly spaced, separated by ridge-free spaces (arrowheads).

Figure 6C (male) six ridges separated by three ridge-free spaces (arrowheads): two ridges mid- dorsal oriented from right to left, one ridge right-ventral, three ridges left-ventral; all these latter with unclear orientation.

Figure 6D (female) seven ridges separated by two ridge-free spaces (arrowheads): one ridge mid-dorsal oriented to left, six ventral ridges (three right-ventral, two left-ventral); tips of these two latter groups divergent.

##### 3.6.1.3 Bursa (number of worms studied not specified, illustrated in [38]: Figs. 30, 33 and 34)

Figure 30: distal extremity including closed bursa, left lateral view, only rays 3-8 illustrated, rays 4-6 joined up to extremities. Figure 33: bursa “partially unrolled in left lateral and dorsal aspects”, only dorsal ray and left rays 4-8 illustrated, extremities of left rays 4-6 diverging. Figure 34: dorsal lobe, orientation not specified. Right lobe not illustrated. From generic definition [38], bursa dissymmetrical with right lobe larger; whereas from description, left lobe larger. From generic definition, pattern of type 1-4.

#### 3.6.2 Comments

##### 3.6.2.1 Head

In the original written description, the cephalic vesicle is indicated as present but is not illustrated. On the other hand, lips and teeth are indicated as absent, but the illustration in apical view shows clearly five lips (Fig. 28 in [38]), which is undoubtedly a drawing flaw since these structures appear in number of six.

##### 3.6.2.2 Synlophe

The position of the ridges and the inclination of the axis are very different between both sexes.

In Figure 6C (male), the tip of the only ridge situated on right-ventral quadrant is perpendicular to the body surface but clearly divergent from the tips of the dorsal ridges. The remaining ventral ridges are also oriented perpendicularly to body surface, there are no convergent tips and the inclination of the axis of orientation remains uncertain.

Figure 6D (female); if the section is reversed on its sagittal axis, then turned about 75° counterclockwise we obtain a section whose synlophe is similar to that of other Heligmosomoidea in which the axis is subfrontal (Fig. 6D’).

Based on these observations, two types of synlophe could be described: Type I (Fig. 6C) characterized by 6 ridges at midbody grouped into three sets with inclination of axis of orientation uncertain. Type II (Fig. 6D’) characterized by 7 ridges at midbody grouped into two sets with inclination of axis of orientation perhaps subfrontal.

The presence of a cephalic vesicle, a developed buccal capsule, and six lips indicate that the specimens studied do not belong to the Heligmonellidae but should rather be assimilated with the Herpetostrongylidae: Herpetostrongylinae (parasitic in reptiles, Australian marsupials and exceptionally in rodents). The Herpetostrongylinae possess the same cephalic characters described above, plus 1-3 oesophageal teeth (not observed by the author in *Rodentanema*). Besides the cephalic characters, the Herpetostrongylinae possess a synlophe with an axis of orientation oblique or subfrontal, bursae of different types including the type 1-4, females didelphic or monodelphic, and female tails with or without a spine.

Among the Herpetostrongylinae, only two genera are characterized by females which are monodelphic and without a caudal spine: *Dessetostrongylus* Humphery-Smith, 1981, parasitic in Dasyuridae (Marsupialia) in Australia, and *Papuastrongylus* Smales, 2010 parasitic in Muridae from Papua New Guinea. The two genera are differentiated from the specimens described as *Rodentanema* by having well-developed buccal capsules and a different synlophe pattern.

##### 3.6.2.3 Bursa

According to [38], Figures 30 and 33 illustrate the left lobe. But both figures are clearly different, particularly concerning left ray 8, which in Figure 33 is seen mostly parallel to ray 6 and in Figure 30 it diverges proximally from it. Figures 30 and 33 correspond clearly to different bursae, which may imply the presence of two different species among the type material. In addition, the right lobe is not illustrated, the description of the bursa is very brief and ambiguous and in the absence of an illustration of ray 2, the pattern cannot be determined.

#### 3.6.3 Conclusion

There seem to be two taxa concerned in the description of this species (each characterized by a different synlophe): “*R. aenigma*” and a Herpetostrongylidae *i.s.* The uncertain orientation of the ridges, the incomplete description of the bursa, plus the fact that we do not know what type of synlophe the holotype corresponds to, *Rodentanema aenigma* is considered a *species inquirenda*. Being the type and unique species of the genus, it is impossible to give a precise definition of it. We thus consider *Rodentanema* a *genus inquirendum*.

### 3.7 Genus *PARVINEMA* Smales, 2017 (Fig. 7)

**Type species:** *Parvinema bafunminense* Smales, 2017.

**Figure 7:**
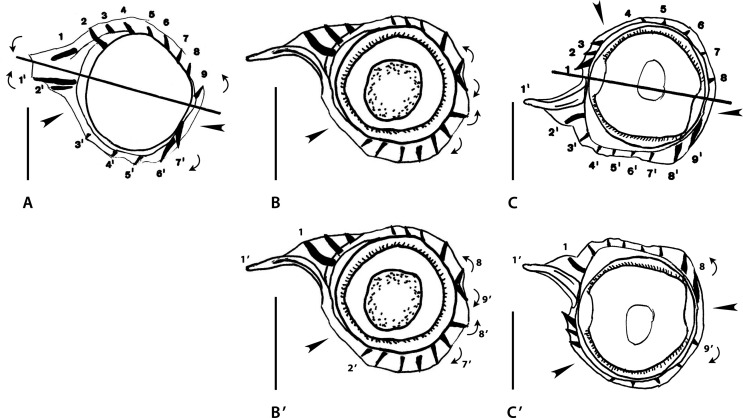
Genus *Parvinema* Smales, 2017. Midbody sections. A, B *Parvinema bafunminense* Smales, 2017. A male, B, B’ female. C, C’ *Parvinema helgeni* Smales, 2017, male. Source: A–C redrawn from [40]. B’, C’ modified figures with respect to the original: B’ numbering of ridges added. C’ reversed on its frontal axis, then rotated 30° clockwise.

**Hosts:** Muridae, Murinae, Hydromyini (Rodentia).

**Host site:** small intestine.

**Distribution:** Papua New Guinea.

**Other species:** *Parvinema helgeni* Smales, 2017 coparasitic with the type species in *Paramelomys lorentzii.*

**Original diagnosis:** *Parvinema* gen. nov. *Heligmonellidae*. *Nippostrongylinae. Synlophe well developed with 15-17 continuous ridges mid body. Carene (sic) present. Left lateral ridges largest, larger than right ridges. Axis of orientation from right ventral to left dorsal. Bursa dissymmetrical, left lobe larger, pattern of bursal rays 1-3-1. Dorsal ray divided distally to branching of rays 8. Parasites of murines. Hydromyini, from New Guinea* [40].

#### 3.7.1 Analysis of data and difficulties encountered

##### 3.7.1.1 *Parvinema bafunminense* (Figs. 7A, 7B)

###### 3.7.1.1.1 Synlophe (Based on sections from six worms, sex not specified)

Sections analyzed herein are at midbody: male (Fig. 18/7A) and female (Fig. 17/7B); lateral cords not illustrated; ridges numbered in Figure 18/7A.

In all sections, careen absent and axis of orientation of ridges described as oblique in [40].

Figure 7A (male): dilatation of cuticle, evoking a careen, illustrated on left side; 16 ridges and two small gaps: one on right-ventral quadrant between ridges 9 and 7’, second one on left-ventral quadrant between ridges 3’ and 2’ (arrowheads); dorsal ridges regularly spaced and similar in size, except ridges 1 and 2, larger; ventral ridges regularly spaced, mid- ventral ones small (5’-3’) and right-ventral ones large (7’, 6’). Despite absence of illustration of lateral cords, tips of ridges oriented from right to left on both sides (dorsal and ventral) with ridge tips 1’ and 1 convergent (curved arrows on the left) and ridges 9 and 7’ divergent (curved arrows on the right), determining an axis of orientation oblique.

Figure 7B (female): left ridge very large, strongly curved (inner curvature downwards); 16 ridges and a large gap on left-ventral quadrant (arrowhead); dorsal ridges irregularly spaced and dissimilar in size, median ones being shortest; ventral ridges mainly right-ventral, regularly spaced and large. On mid-right side, pair of divergent tips present and, immediately ventral, another pair of divergent ridges (curved arrows); dorsal and ventral ridges oriented from right to left.

###### 3.7.1.1.2 Bursa (number of worms studied not specified, illustrated in [40]: Figs. 21, 25 and 26)

Figure 21: distal part of dorsal ray and rays 8, orientation not specified. Figure 25: left lateral lobe, orientation not specified, only rays 3-6 illustrated, no link with left ray 8. Figure 26: right lateral lobe, orientation not specified, right ray 8 also illustrated. From the written description: bursa dissymmetrical with left lobe larger and pattern of type 1-3-1.

##### 3.7.1.2 *Parvinema helgeni* (Fig. 7C)

###### 3.7.1.2.1 Synlophe (based on section of one male)

Section analyzed herein is at midbody (Fig. 31/7C); lateral cords illustrated; ridges numbered. Careen absent. Ridge 1’ very large and slightly curved (inner curvature upwards). Sixteen ridges irregularly spaced with two gaps (arrowheads): one between ridges 3 and 4, another one between 8 and 9’. Two groups of dorsal ridges: on left three small ridges, serried, oriented from right to left; on right, five small ridges, more widely spaced, oriented perpendicular to body surface. Ventral ridges regularly spaced with median ridges smallest, oriented from right to left. Axis of orientation oblique.

###### 3.7.1.2.2 Bursa (illustrated in [40]: Fig. 32)

Figure 32: dorsal ray, left ray 8 and left rays 4-6, left latero-dorsal view; rays 2 and 3 not illustrated. From the written description [40]: bursa dissymmetrical with left lobe larger and pattern of type 1-3-1.

#### 3.7.2 Comments

##### 3.7.2.1 Synlophe

Although in the definition of the genus [40] a careen is mentioned, in the written descriptions of *P. bafunminense* and *P. helgeni*, there is no reference to such a structure. Illustrations of *P. bafunminense* show some flaws: in Figure 7A, ridge 1 does not reach the margin of the section, and the dorsal hypodermis is not correctly illustrated (present on the left, absent on the right).

The male section of *P. bafunminense* is clearly different from the female section of the same species and from that of *P. helgeni* by the ridge 1’ not very large and straight.

In the male section of *P. helgeni*, the orientation of ridge 1’ is unusual for a Nippostrongylinae and it is likely that the section should be reversed on its frontal axis (Fig. 7C’). After reversion and a slight rotation clockwise, the section becomes similar to that of *P. bafunminense* female at midbody: large left ridge oriented with the inner curvature downwards; dorsal ridges regularly spaced and unequal in size, the mid-dorsal ones being small and the left-dorsal (1, 2) and right-dorsal ones (6 to 8) being larger. On the reoriented section the dorsal ridges are oriented from right to left; the ventral ridges 9’ to 5’ oriented perpendicularly to body surface and left ventral ridges (4’ to 1’) oriented from right to left.

The synlophes of *P. bafunminense* female and *P. helgeni* male share the left ridge (ridge 1’) very large and curved and a left-ventral gap. However, in *P. bafunminense* female, the gap is situated between ridges 1’ and 2’ whereas in *P. helgeni* male, it is situated between ridges 5’ and 4’. In both species the inclination of the axis of orientation is uncertain due to the disparate orientation of the ridges.

The male and the female of *P. bafunminense* do not appear to belong to the same taxon, the differences between both synlophes being too marked. The synlophe of *Parvinema bafunminense* (female) is closely related to that of *P. helgeni* (male) by the hypertrophy of the left ridge and they should probably be included in the same genus. They differ from each other by the relative size and spacing of the gaps, characters which could be attributed to specific differences.

##### 3.7.2.2 Bursa

*Parvinema bafunminense*: Figure 21, judging from the shape of the bursa, is in dorsal view. In Figures 25 and 26 the margins are illustrated with dotted lines, which indicates that the lateral lobes are in latero-dorsal view. Judging from the illustrations, the pattern is 2-2-1 in both lobes, even if left ray 2 is not illustrated. *Parvinema helgeni*: the unique drawing provided is incomplete and does not allow us to confirm or to dismiss either the dissymmetry or the pattern 1-3-1 of the bursa.

#### 3.7.3 Conclusion

It is possible that under *Parvinema* there are at least three taxa described. This hypothesis is reinforced by the fact that in [40] it is noted that “*Parvinema helgeni* and *P. bafunminense* were found in mixed infections in four of the 27 individuals of *P. lorentzii* examined” (p. 770).

Unfortunately, the original description did not provide more precision. For *P. bafunminense* it is not possible to know if the description and illustration of the bursa come from the same individuals used for the study of the synlophe. On the other hand, the female synlophe of *P. helgeni* is not described nor illustrated. In these conditions, *Parvinema bafunminense* and *Parvinema helgeni* are considered species *inquirendae* and the genus *Parvinema* a *genus inquirendum*.

The three types of synlophe recognized seem to be distributed into three different taxa, as follows: “*P. bafunminense*” (male section), Nippostrongylinae *i.s.* 4 (female section of *P. bafunminense*), and “*P. helgeni*” (male section).

### 3.8 Genus *MISSIMSTRONGYLUS* Smales, 2018 (Fig. 8)

**Type species***: Missimstrongylus oweni* Smales, 2018.

**Figure 8:**
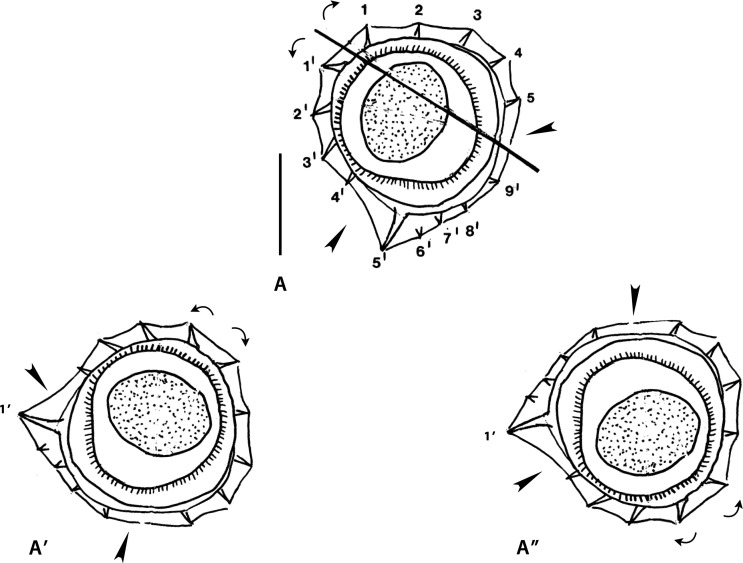
Genus *Missimstrongylus* Smales, 2018. A-A” *Missimstrongylus oweni* Smales, 2018, section at midbody, male. Source: A redrawn from [41]. A’, A” modified figures: A’ rotated 90° clockwise with respect to the original, A” further reversion of A’ on its frontal axis.

**Hosts:** Muridae, Murinae (Rodentia).

**Host site:** small intestine.

**Distribution:** Papua New Guinea.

**Original diagnosis:** *Nippostrongylinae: Synlophe with continuous ridges, 14 in mid body male, ridges unequal in size, ridge 5’ largest, axis of orientation of ridges oblique, from ventral right to dorsal left, lacking a carene (sic). Bursa dissymmetrical, left lobe larger, dorsal lobe shorter than lateral lobes, bursal pattern 1-3-1. Spicule to body length ratio 18-19%. Parasites of murines, Rattini, from Papua New Guinea* [41].

#### 3.8.1 Analysis of data and difficulties encountered *Missimstrongylus oweni*

##### 3.8.1.1 Synlophe (number of worms studied not specified)

Section analyzed herein is at midbody, male (Fig. 2/8A). Lateral cords not illustrated; ridges numbered.

Careen absent. Fourteen ridges small, except ventral ridge 5’ medium sized, and ridges 8’-6’ minute. Two small gaps (arrowheads) on left-ventral left side between ridges 5’ and 4’ and on right-right ventral side between ridges 5 and 9’. Tips of ridges 1’ and 1 divergent (curved arrows). Ridges 1’ to 4’ oriented from dorsal to ventral, ridges 1 to 5 oriented from left to right, ridge 5’ oriented from right to left, remaining ventral ridges, oriented perpendicularly to body surface. Axis of orientation described as oblique in [41].

##### 3.8.1.2 Bursa (number of worms studied not specified; illustrated in [41]: Figs. 4 and 7)

Figure 4, “left lateral view”, orientation not specified, rays 2-9 illustrated but no link between rays 6 and 8. Figure 7, “right lateral view”, orientation not specified, only lateral and ventral rays illustrated. From diagnosis [41]: bursa dissymmetrical with right lobe larger and pattern of type 1-3-1; from the written description, bursa with left lobe larger.

#### 3.8.2 Comments

##### 3.8.2.1 Synlophe

The ridge arrangement illustrated in [41] does not match the usual orientation of the ridges in the Nippostrongylinae.

If the section is turned 90° clockwise (Fig. 8A’), the largest ridge is placed on the left (a common arrangement in the Nippostrongylinae). But, in doing so the divergent ridge tips are situated in the right dorsal-quadrant (never found in the Nippostrongylinae). A further reversion of the section on its frontal axis (Fig. 8A”) keeps the largest ridge on the left and places the divergent ridges in the right-ventral quadrant. However, in doing so, the dorsal ridges become oriented rather anarchically (right-dorsal ones from left to right and left-dorsal ones perpendicular) and the largest ridge on the left is pointing to the ventral side, an orientation never found in the Nippostrongylinae. This synlophe does not actually match any other in the subfamily and no manipulation of the section (rotation and/or reversion) will make the synlophe to match the usual orientation observed in the Nippostrongylinae.

##### 3.8.2.2 Bursa

The expressions “left lateral view” or “right lateral view” concerning the bursa are ambiguous since they may actually refer to either lateral lobe (left or right) on either of their two surfaces (dorsal or ventral). Anyway, we assume that Smales [41] intended to illustrate the left lobe in Figure 4 and the right lobe in Figure 7. The right lobe is clearly longer than the left lobe. Judging from Figure 7, the left and right lobes are in dorsal view. The pattern is 1-4 for both lobes; right lobe with ray 3 diverging at same level as ray 6, rays 4 and 5 parallel and joined to their extremities; left lobe with ray 3 diverging proximally to ray 6, rays 4-6 parallel and joined to their extremities.

#### 3.8.3 Conclusion

In her *Remarks*, Smales [41] relates *Missimstrongylus* to *Hasanuddinia*, *Melomystrongylus*, *Nugininema* and *Montistrongylus*, all of which have synlophes with large ventral ridges. However, the data provided in the description of the type species of *Missimstrongylus* are insufficient to assert that the most developed ridge is indeed ventral in position. It could be also the left ridge. Under these conditions, *Missimstrongylus oweni* is considered a *species inquirenda* and, consequently, *Missimstrongylus* a *genus inquirendum*.

### 3.9 Genus *FLANNERYSTRONGYLUS* Smales, 2019 (Fig. 9)

**Type species:** *Flannerystrongylus abulus* Smales, 2019.

**Figure 9:**
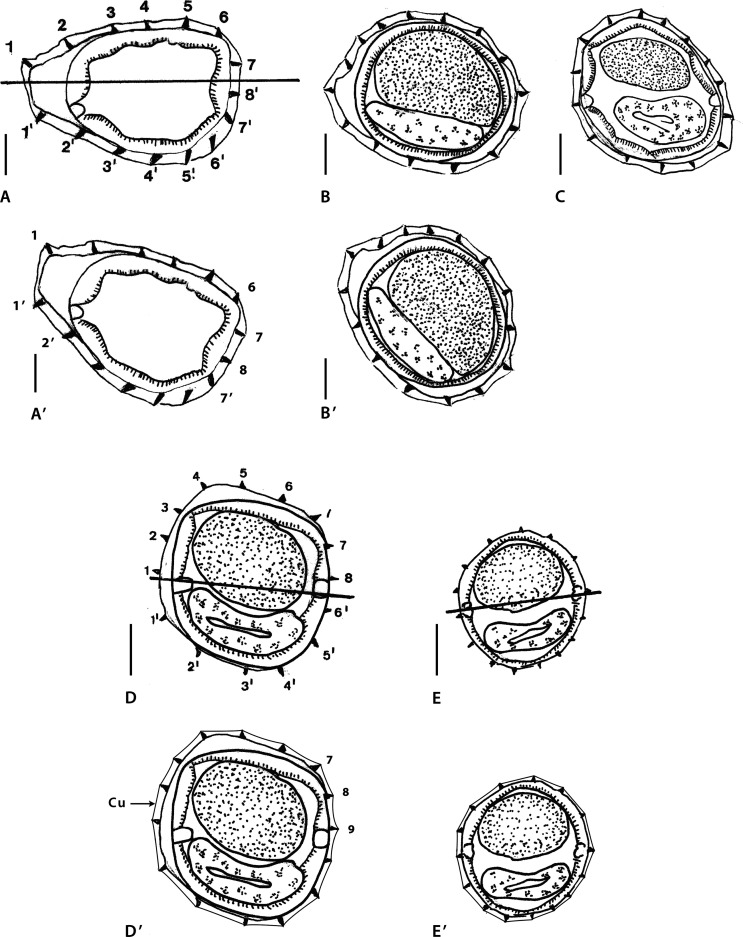
Genus *Flannerystrongylus* Smales, 2019. Body sections. A–C *Flannerystrongylus abulus* Smales, 2019. A, A’ within proximal body, female. B, C at midbody. B male, C female. D–E’ *Flannerystrongylus chisholmae* Smales, 2020. D–E’ at midbody. D, D’ male. E, E’ female. Abbreviation: cu, cuticle. Sources: A–C redrawn from [42]; D, E redrawn from [43]. A’, B’, D’, E’: modified figures: A’ rotation clockwise, re-numbering of ridges with respect to the original. B’ rotation clockwise. D’, E’ addition of external cuticular lining. D’ re-numbering of ridges.

**Hosts:** Muridae, Murinae (Rodentia).

**Site:** small intestine.

**Distribution:** New Guinea.

**Other species:** *Flannerystrongylus chisholmae* Smales, 2020.

**Original diagnosis:** *Heligmonellidae, Nippostrongylinae. Synlophe well developed with 14-16 continuous, pointed, evenly-sized, longitudinal ridges; axis of orientation of ridges sub-frontal anteriorly. Bursa dissymmetrical, left lobe larger. Pattern of bursal rays 2-3, dorsal ray divided at level of branching of rays (8) from dorsal trunk. Parasites of uromyin murids* [42].

#### 3.9.1 Analysis of data and difficulties encountered

##### 3.9.1.1 *Flannerystrongylus abulus* (Figs. 9A–9C)

###### 3.9.1.1.1 Synlophe (based on sections from ten specimens; sex not specified)

Sections analyzed herein are within proximal body: female (Fig. 2/9A) and at midbody: male (Fig. 6/9B) and female (Fig. 5/9C); lateral cords illustrated in Figures 2/9A (left cord) and 2/9 C; ridges numbered in Figure 2/9A.

In all sections, careen absent. Axis of orientation subfrontal within proximal body, not specified at midbody [42].

*Within proximal body:* in Figure 9A (female), 15 ridges regularly spaced and subequal in size; on dorsal side, ridges 1-4 and 6 oriented from right to left, ridges 5 and 7, perpendicular to body; on ventral side, all ridges oriented from right to left except ridge 8’ perpendicular to body; axis of orientation not passing through left lateral cord (right one not illustrated) but above and starting between ridges 7 and 8’ whose tips are not divergent, then passing between ridges 1 and 1’ whose tips are not convergent but divergent.

*At midbody:* in Figures 9B (male) and 9C (female), 15 ridges regularly spaced and subequal in size, oriented disparately.

###### 3.9.1.1.2 Bursa (number of worms studied not provided, illustrated in [42]: Figs. 12, 14 and 15)

Figure 12: left lateral view of one lobe (not specified), ventral rays situated towards bottom of page, only distal part of rays 2-8 illustrated, ray 8 joined proximally to ray 6. Figure 14: dorsal lobe, view not specified, only distal extremity of dorsal ray and rays 8 illustrated, both strongly curved, one of them extending beyond extremity of dorsal ray. From the written description: rays 8 diverge at level of division of dorsal ray, *i.e.*, at one-third of length; left ray 8 larger. Figure 15: right lateral view of one lobe (not specified), ventral rays situated towards bottom of page; one branch of dorsal ray and distal part of rays 2-8 illustrated; ray 8 slightly curved and reaching level of extremity of dorsal ray; ray 6 straight, rays 4-5 apparently curved dorsally.

From the written description [42]: bursa dissymmetrical with left lobe larger; lateral rays 6 curved dorsally, rays 4, 5 ventrally; rays 4 largest; rays 2-3 diverge ventrally, reaching margin of bursa; pattern of type 2-3.

##### 3.9.1.2 *Flannerystrongylus chisholmae* (Figs. 9D–9E')

###### 3.9.1.2.1 Synlophe (based on sections from two males and five females)

Sections analyzed herein are at midbody male (Fig. 2C/9D) and female (Fig. 2E/9E). In both sections lateral cords illustrated, and ridges numbered in Figure 2C/9D.

In both sections careen absent; 15 minute ridges regularly spaced and subequal in size; axis of orientation described as subfrontal by Smales [43] but no arrow indicating its direction. In the original figures, the external lining of cuticle that connects the ridges to each other is not drawn.

Figure 9D (male): on dorsal surface, non-numbered ridge between ridges 6 and 7; some ridges pointing to left, some others to right and remaining oriented perpendicularly to body surface in rather disparate arrangement.

Figure 9E (female): ridges mostly oriented perpendicularly to body surface.

###### 3.9.1.2.2 Bursa (number of worms studied not specified, illustrated in [43]: Figs. 2G, 2L and 2N)

Figure 2G, left lobe, view not specified, rays 2-6 numbered, only distal part of rays illustrated. Figure 2L, dorsal lobe, view not specified, only distal extremity of dorsal ray and rays 8 illustrated. Figure 2N, right lobe, view not specified, ray 6 smaller and separated from rays 4-5. From the written description [43]: bursa dissymmetrical with left lobe larger, lateral rays 4-6 about same length, reaching margin of bursa, pattern of type 2-3.

#### 3.9.2 Comments

##### 3.9.2.1 Synlophe

*Flannerystrongylus abulus:* within the proximal part of the body, the female section (Fig. 9A) should be slightly rotated clockwise to align the left lateral cord horizontally and to have the accurate orientation of the section (Fig. 9A’). We propose a slight modification on the numbering of the ridges, so that ridge 8’ in [42] becomes ridge 8. Figure 9B can be similarly rotated clockwise (Fig. 9B’). This re-arrangement would make all three synlophes (A’, B’, C) more or less consistent, with an oblique axis of orientation separating 8 right-dorsal and 7 left-ventral ridges although passing between incorrectly oriented ridges.

*Flannerystrongylus chisholmae:* on the male section (Fig. 9D) ridge 7 in [43] is in fact ridge 8, and ridge 8 in [43] becomes ridge 9 (Fig. 9D’). In both sections the external lining of the cuticle must be added (Figs. 9D’ and 9E’).

In both species, at midbody, an axis of orientation of the ridges cannot be identified in any of the sections because most ridges are oriented perpendicularly to the body surface.

In her comments, Smales [42] gives a rather detailed differential diagnosis against 41 genera from the Sahul region and Malaysia pointing that *Flannerystrongylus* is characterized by the absence of a gradient in ridge size and by a regular spacing of the ridges, this latter character being relatively rare in this group of Australasian genera. Both elements, subequal ridges and spaced regularly are found in the genus *Equilophos*, considered by Smales [42] as the most related morphologically, differing mainly from *Flannerystrongylus* by the number of ridges (more than 30 in *Equilophos vs.* 15 in *Flannerystrongylus*).

##### 3.9.2.2 Bursa

*Flannerystrongylus abulus:* the positions of Figures 12 and 15 in [42] do not follow the rules generally agreed in Zoology, *i.e.*, that the animal should be drawn with the proximal part to the top of the page, which makes the comparison with other species easier. The expressions “left lateral view” or “right lateral view” concerning the bursa are ambiguous since they may actually refer to either lateral lobe (left or right) on either of their two surfaces (dorsal or ventral). It is based on the curvature of the lateral rays that we deduce that Figure 12 corresponds to the left lobe in ventral view and Figure 15 to the right lobe also in ventral view. From the written description, Figure 14 in [42] is in dorsal view since the left ray 8 is said to be larger than the right one.

Judging from the figures, there are three different arrangements of rays 8 with respect to the dorsal ray and the lateral lobes: (1) that on Figure 12, with left ray 8 diverging from the common trunk of rays 2-6; (2) that on Figure 14, with left ray 8 curved diverging from the dorsal ray just above the division of this latter and completely separated from ray 6, right ray 8 strongly curved, touching distally the right branch of the dorsal ray; (3) that on Figure 15, with right ray 8 not strongly curved, distant from the right branch of the dorsal ray.

Figure 14 matches the written description, whereas Figures 12 and 15, for different reasons, do not. We are not able to know if Figure 12 corresponds to another type of bursa, because the description provided is insufficient. The same observation applies to Figure 15, in which, for instance, the divergence of rays 2 and 3 is not observed. This may imply the presence of two or three different species among the type material. These bursae have a pattern of type 2-2-1.

*Flannerystrongylus chisholmae:* despite the incomplete description of the bursa, the pattern is clearly 1-4 on both lobes. On the left lobe, ray 3 diverges proximally to ray 6, this latter being joined to rays 4 and 5 up to their extremities. On the right lobe, rays 3 and 6 diverge at the same level from the common trunk of rays 3-6 and rays 4 and 5 are joined up to their extremities. In [43], Figure N shows the right ray 6 much shorter than right rays 4 and 5, in contradiction with the written description. Figures G and L match the written description, whereas Figure N does not.

#### 3.9.3 Conclusion

In view of the very brief written description, the many problems with the illustrations of the synlophe, and the contradictory illustrations of the bursa, *Flannerystrongylus abulus* is considered a *species inquirenda* and, consequently, *Flannerystrongylus* a *genus inquirendum*. *Flannerystrongylus chisholmae* is also considered a *species inquirenda* due to its insufficient description.

### 3.10 Genus *HELGENEMA* Smales, 2020 (Fig. 10)

**Type species:** *Helgenema keablei* Smales, 2020.

**Figure 10:**
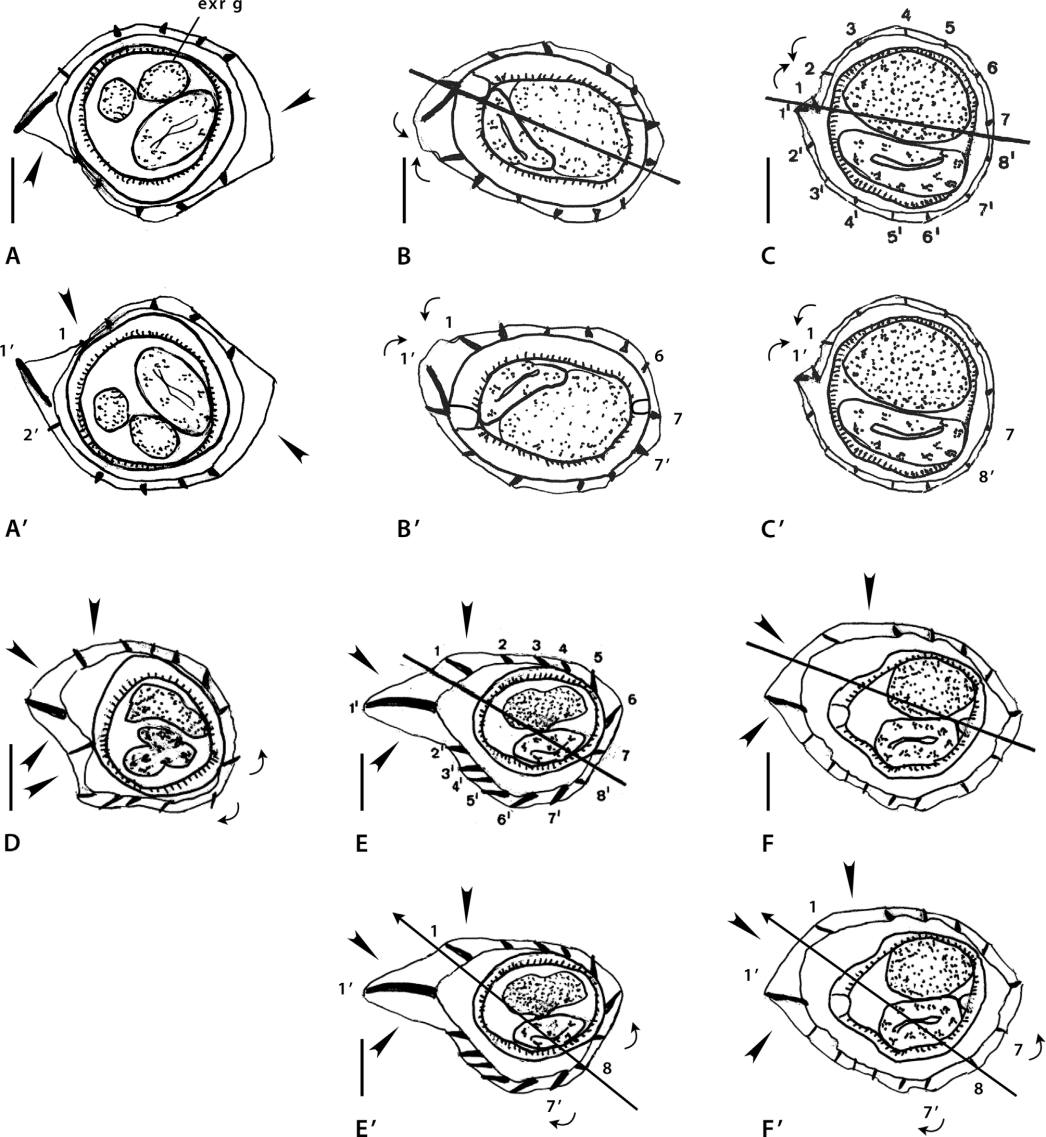
Genus *Helgenema* Smales, 2020. Body sections. A–C’ *Helgenema keablei.* A, A’ within proximal body, female. B–C’ at midbody. B, B’, male. C, C’ female. D–F’ *Helgenema lamia* Smales, 2021*.* D within proximal body, male. E–F’ at midbody. E, E’ male, F, F’, female. Sources: A–C redrawn from [43]. D–F redrawn from [44]. A’–C’, E’, F’ modified sections: A’ reversed on its frontal axis with respect to the original, B’ reversed on frontal axis then rotated *ca*. 15° clockwise with respect to the original. C’, E’, F’ re-numbering of ridges.

**Hosts:** Muridae, Uromyinae (Rodentia).

**Host site:** small intestine.

**Distribution:** Papua New Guinea.

**Other species:** *Helgenema lamia* Smales, 2021.

**Original diagnosis:** *Heligmonellidae, Nippostrongylinae. Synlophe well developed with 11-15 pointed longitudinal continuous ridges. Cuticle with dilatation on left side supported anteriorly by large left dorso-lateral ridge. Axis of orientation sub frontal. Bursal pattern 2-3. Dorsal ray divided within distal half. Spicule to body length ratio 7.2%. Parasites of uromyin murids* [43].

#### 3.10.1 Analysis of data and difficulties encountered

##### 3.10.1.1 *Helgenema keablei* (Figs. 10A–10C’)

###### 3.10.1.1.1 Synlophe (based on sections from two males and two females)

Sections analyzed herein are within proximal body: female (Fig. 3B/10A), and at midbody: male (Fig. 3A/10B) and female (Fig. 3E/10C). Lateral cords illustrated in Figure 3E/10B, ridges numbered in Figure 3E/10C.

In all sections, careen absent. Axis of orientation of ridges described as subfrontal in [43].

*Within proximal body:* in Figure 10A (female), 11 ridges irregularly spaced, clearly separated into two groups (6 dorsal, 5 ventral) by two lateral gaps, one left- ventral, one mid-right (arrowheads); ridges small and subequal except left ridge, large, oriented towards ventral side; most ridges within each group disparately oriented; within section, two structures resembling excretory glands figured dorsally with respect to intestine.

*At midbody:* in Figure 10B (male), 13 ridges, regularly spaced and unequal in size; in front of left lateral cord, large ridge oriented towards ventral side and convergent with ridge ventrally adjacent (curved arrows), supporting small cuticular dilatation; remaining ridges small, disparately oriented.

Figure 10C (female): 15 ridges regularly spaced, subequal in size and small, including ridges 1’ and 1; ridges numbered from 1 to 7 dorsally and from 1’ to 8’ ventrally; ridges numbered as 1 and 2 apparently convergent (curved arrows), most of remaining ridges perpendicular to body surface.

###### 3.10.1.1.2 *Bursa* (number of worms studied not specified, illustrated in [43]: Figs. 3I, 3J and 3K)

Figure 3I: left lobe, orientation not specified, with no link to left ray 8. Figure 3J, dorsal lobe, orientation not specified with no link to rays 6, rays 8 arising just proximally to division of dorsal ray and reaching level of extremities of this latter. Figure 3K: right lobe, orientation not specified, with no link with right ray 8. From the written description [43] rays 8 not described, rays 4-6 about same size, reaching margin of bursa, bursa dissymmetrical with left lobe larger and pattern of type 2-3.

##### 3.10.1.2 *Helgenema lamia* (Figs. 10D–10F’)

###### 3.10.1.2.1 Synlophe (based on sections from two males and two females)

Sections analyzed herein are within proximal body: male (Fig 1b/10D), and at midbody: male (Fig. 1f/10E) and female (Fig. 1a/10F); lateral cords illustrated in Figure 1a/10F; ridges numbered in Figure 1f/10E.

In all sections careen absent and left cuticular dilatation present. Axis of orientation described as oblique (from right-ventral to left-dorsal) in [44].

*Within proximal body:* in Figure 10D (male), 14 ridges irregularly spaced, unequal in size. Four gaps on left side (arrowheads); ridges medium sized and subequal, except one left ridge, large. Ridges oriented in two opposite directions from right-ventral quadrant to left, starting from a pair of divergent ridges (curved arrows) but not clear convergent ridges on left-dorsal quadrant; the two ridges flanking left ridge (dorsally and ventrally) slightly larger than remaining ridges and perpendicular to body surface.

*At midbody:* in both sexes, 15 ridges irregularly spaced and unequal in size.

Figure 10E (male): three gaps on left side (arrowheads) between ridges 2’ and 1’, 1’ and 1, 1 and 2; ridge 1’ large, ridge 1 medium-sized; other dorsal ridges small, regularly spaced and oriented from right to left, with ridges 5 and 6 larger; ventral ridges medium sized, regularly spaced and oriented from right to left, with ridges 6’ and 7’ larger and ridge 8’ minute; axis of orientation drawn in [44] passes between ridges 7 and 8’ (whose tips not divergent) then between ridges 1’ and 1.

Figure 10F (female): three gaps on left side (arrowheads); left ridge largest; first dorsal ridge on the left larger than other dorsal ridges, small and regularly spaced. On ventral side, ridges small, subequal in size and much thinner than in male section. Dorsal and ventral ridges disparately oriented.

###### 3.10.1.2.2 Bursa (illustrated in [44]: Fig. 1L)

Figure 1L in captions (corresponds to “i” on Fig. 1). Entire bursa with left lobe folded ventrally, dorsal view. Bursa dissymmetrical with left lobe larger. From the written description [44], bursal pattern of type 2-3.

#### 3.10.2 Comments

##### 3.10.2.1 Helgenema keablei

*Synlophe:* from the original description [43], the synlophe within the proximal region possesses 13-14 ridges in one male, 13 ridges in another male, 9-11 in one female, 11-15 in another female; and, the synlophe at midbody 14 ridges in males, 15 in females. Variation in ridge number from nine to 15 within the proximal body seems to us unlikely, and we assume that the “proximal” sections in different specimens have not been taken at homologous levels, the sections with more ridges having been probably taken closer to midbody.

The section in 10A should be reversed on its frontal axis, to match the usual arrangement of the Nippostrongylinae. In the re-oriented section (Fig. 10A’) the excretory glands are situated ventrally with respect to the excretory pore, and the large left ridge can be interpreted as ridge 1’, with its tip pointing dorsally, allowing the numbering of the remaining ridges according to the usage in the Nippostrongylinae.

Concerning the midbody male section (10B), the position of the right lateral field is unlikely because a hypothetical frontal axis passing through the illustrated fields would determine a ventral part much larger than the dorsal one. We propose to displace the right lateral field up to the axis originally illustrated in [43] in order to have two equivalent dorsal and ventral parts. We think that this section should be reversed on its frontal axis so that the large left ridge, which we interpret as ridge 1’, is directed dorsally. We propose a further rotation (*ca*. 15°) of the re-oriented section so that the new axis of orientation passes, on the left, between tips of the ridges 1 and 1’ which are clearly convergent. On the right-ventral side the ridges are oriented perpendicularly to the body, which does not allow us to determine the start of the axis. However, given the position of the ridges 1 and 1’, it is probably oblique (Fig. 10B’).

In the midbody female section (10C) the tips of the ridges 1 and 2 being convergent, the numbering must be modified and ridge 1 becomes ridge 1’ and ridge 2 becomes ridge 1 (Fig. 10C’). For the same reasons than in the male, the axis of orientation is possibly oblique.

##### 3.10.2.2 Helgenema lamia

*Synlophe*: in the proximal male section 10D, the large left ridge is interpreted as ridge 1’. This ridge numbering conforms that of the male section at midbody (Fig. 10E).

At midbody, the only clearly divergent ridges are 8 and 7’ (curved arrows, Fig. 10E’) rather than 7 and 8’ (Fig. 10E) as proposed in [44].

In the three sections, the axis of orientation is possibly oblique but its inclination is calculable only in the female section at midbody, the lateral cords being illustrated (Fig. 10F’).

#### 3.10.3 Conclusion

##### 3.10.3.1 Synlophe

Even if the modifications proposed herein are right, it is not possible to know how the section 10A’ (proximal synlophe of *H. keablei*, female) is related to the midbody female section (10C’) or to the midbody male section (10B’). A large left ridge is present within the proximal female section (10A’) and it is found again in the midbody male section (10B’). On the contrary, the midbody female section (10C’) shows all ridges small and subequal and the large left ridge is absent. Sections A’ and B’ may then be assumed as corresponding to the same synlophe. This synlophe would be similar to that of *Pogonomystrongylus*, where a large left ridge is observed all along the body. On the contrary, there are no examples of large left ridges well-developed proximally and decreasing in size towards midbody. That is why it is difficult to assume sections 10A’ and 10C’ correspond to the same synlophe.

The original definition of the genus [43] indicated that the main character separating *Helgenema* from the remaining genera was the “*Cuticle with dilatation on left side supported anteriorly by large left dorso-lateral ridge*”. Some of the sections analyzed herein contradict this definition in several points: (1) the large ridge is actually left or left-ventral (ridge 1’) and not dorsal, (2) the midbody male section of *H. keablei* shows the dilatation supported by *two* ridges, (3) the proximal male section of *H. lamia* does not show an apparent cuticular dilatation, and (4) the midbody male section of *H. lamia* shows a large ridge 1’ which clearly is more developed than at proximal body. This latter condition cannot even be confirmed in the female of the same species, since the synlophe at proximal body is not provided.

We think that among the material concerned there are two types of synlophe: one with a large left ridge, more developed than the other ridges, all along the body (sections 10A’ and 10B’ of *Helgenema keablei* and 10D–10F of *Helgenema lamia*). The other one, without large left ridge, with ridges small and subequal (section 10C of *Helgenema keablei*)*.*

##### 3.10.3.2 Bursa

In both, *Helgenema keablei* and *H. lamia*, since rays 2 diverge first from the trunk 2-6, the bursal pattern is of type 1-4.

#### 3.10.4 Conclusion

Without indication about the exact level of the sections, without the illustration of both sexes at the proximal part, and without precision on the material studied for the female sections (on same or different specimens), *Helgenema keablei* and *Helgenema lamia* are considered *species inquirendae* and *Helgenema* a *genus inquirendum*. It would be possible that under *Helgenema* there are two or three different taxa, with the two types of synlophe recognized distributed as follows: “*H. keablei*” (midbody male section and proximal female section); “*H. lamia*” (male and female); Nippostrongylinae *i.s*. 5 (midbody female section of *H. keablei*).

### 3.11 Genus *PARAMELOMYSTRONGYLUS* Smales, 2020 (Fig. 11)

**Type and sole species:** *Paramelomystrongylus dessetae* Smales, 2020.

**Figure 11:**
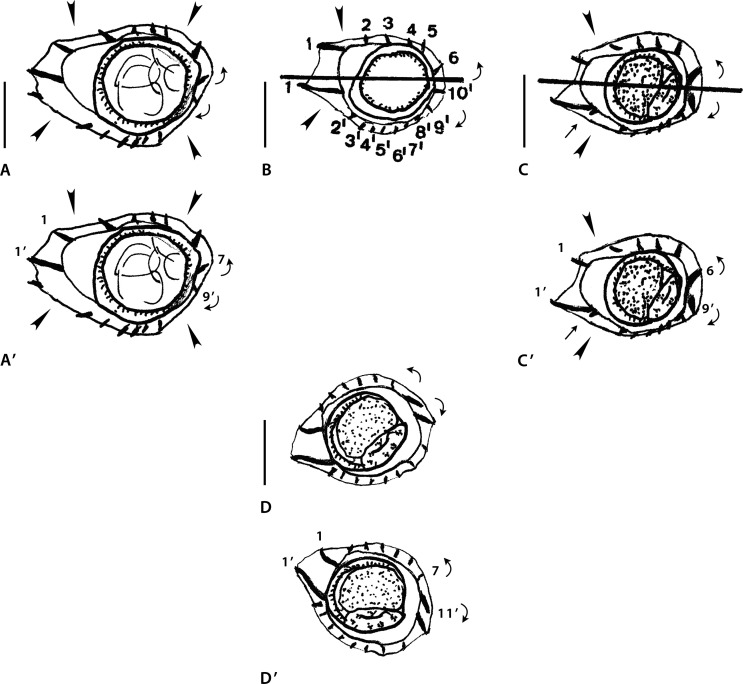
Genus *Paramelomystrongylus* Smales, 2020. Body sections. A–D’ Paramelomystrongylus dessetae Smales, 2020. A, A’ within proximal body, female. B at midbody, male. C, C’ within distal part, female. D, D’ at midbody, female. A, B, C: Synlophe of type I. D: Synlophe of type II. Source: A–D redrawn from [43]. A’, C’, D’ modified sections: A’, C’ numbering of ridges. D’ rotation clockwise and numbering of ridges with respect to the original.

**Hosts:** Muridae, Murinae, Hydromyini (Rodentia).

**Host site:** small intestine.

**Distribution:** New Guinea.

**Original diagnosis:** Paramelomystrongylus *new genus*: *Heligmonellidae, Nippostrongylinae. Synlophe well developed, with 13-16 pointed longitudinal ridges, dorsal ridges continuous, ventral ridges disrupted. Carene supported by 2 hypertrophied ridges, left ventral ridge largest; ridges unequal in size, ridges supporting carene* (sic) *and ridges associated with right lateral side largest. Axis of orientation of ridges sub frontal. Bursal pattern 2-3. Dorsal ray divided within distal half. Spicule to body length ratio 15%. Parasites of uromyin murids* [43]*.*

#### 3.11.1 Analysis of data and difficulties encountered *Paramelomystrongylus dessetae*

##### 3.11.1.1 General

There are two numbering mistakes in [43]. In Figure 8, the reference “D” is employed twice, whereas “G” is absent from the plate (though present in the Captions). We interpret therefore that captions E, F, G (which are successive in the Captions) correspond to body sections D, E and F, which are successive in the plate (proximal, mid-body and distal), respectively. On the other hand, caption “G” refers to a female “posterior section midbody transverse section”. We assume that it concerns a section within the distal part of the body. Due to this labelling confusion of the original, we only use herein our own numbering for the body sections.

##### 3.11.1.2 Synlophe (based on sections from two males and two females)

Sections analyzed herein are those within proximal body: female (Fig. 11A), and at midbody: male (Fig. 11B) and female (Fig. 11D); within distal part of female (Fig. 11C). No lateral hypodermal cords shown in any section; ridges numbered in Fig. 11B.

In all sections, careen present supported by two ridges of which ventral one largest. In Figures 11A and 11C ventral ridges exceed edge of external line of cuticle except ridge 2 of Figure 11C which does not reach this latter. From Smales [43], the dorsal ridges are continuous, the ventral ones discontinuous, and the axis of orientation is described as subfrontal.

*Within proximal body:* in Figure 11A (first female section in [43]), 16 ridges; ridges irregularly spaced with four gaps: two on left side and two on right side (arrowheads); dorsal ridges medium- to small-sized, oriented from right to left; six ridges grouped mid-ventrally, regularly spaced, subequal and small, oriented from right to left. On right side, two divergent ridges (curved arrows), of which ventral one small.

*At midbody:* in Figure 11B (male), 16 ridges irregularly spaced with two gaps, one on each side of careen (arrowheads); dorsal and ventral ridges small and regularly spaced, ventral ones more serried than dorsal ones. Ridge 10’ oriented perpendicularly to body surface. Ridges 6 and 9’ oriented in opposite directions (curved arrows).

Figure 11D (second female section in [43]): 18 ridges including careen. Presence of 2 large ridges opposite to careen, strongly directed ventrally and clearly diverging with respect to dorsal ridges (curved arrows). Remaining ridges small, regularly spaced, mainly oriented perpendicularly to body surface.

*Within distal body*: in Figure 11C (third female section in [43]), 15 ridges irregularly spaced with two gaps, one on each side of careen (arrowheads); dorsal and ventral ridges regularly spaced, dorsal ridges medium-sized, ventral ridges smaller and more serried than dorsal ones; presence of minute ridge at base of ventral ridge of careen (straight arrow). Opposite to careen, tips of two ridges pointing in opposite directions (curved arrows).

##### 3.11.1.3 Bursa (illustrated in [43]: Figs. 8I, 8J, 8K)

Figure 8I, left lobe, orientation not specified, with no link with left ray 8, rays 2-6 illustrated. Figure 8J, dorsal lobe, orientation not specified, with no links to rays 6. Figure 8K, right lobe, orientation not specified, rays 2-6 and right ray 8 illustrated. From the text [43], the bursa is dissymmetrical with left lobe larger, dorsal lobe is shorter than lateral ones, pattern of type 2-3.

#### 3.11.2 Comments

##### 3.11.2.1 Synlophe

In all figures, the presence of a careen allows the numbering of the ridges (Figs. 11A’ and 11C’). The presence of: (1) careen, (2) two groups of ridges (dorsal and ventral) pointing in opposite directions, and (3) right ridges with divergent tips, allow us to determine an axis of orientation that is the same in all sections. In the absence of the lateral cords as a reference, the inclination of this axis with respect to the sagittal axis remains uncertain. For the other characters, three sections are similar: female within proximal body (11A’), male at midbody (11B) and female within distal body (11C’).

In Figure 11D, the two large ridges opposite the careen are considered as right ridges. They are strongly directed ventrally and clearly diverge with respect to the dorsal ridges. This could indicate the start of an oblique axis of orientation, on the right side, inclined from right-dorsal to left-ventral. Such an inclination has never been observed among the Nippostrongylinae, and we propose that Figure 11D be slightly rotated clockwise so that this axis becomes perpendicular to the sagittal axis (Fig. 11D’). However, on the left side the careen is determining another axis with a different inclination, oblique from right-ventral to left-dorsal side. The result is a double axis whose inclination has never been observed because usually, among the Nippostrongylinae, the left axis is more inclined on the sagittal axis that the right one. In addition, the orientation of the remaining ridges is uncertain.

Under *Paramelomystrongylus* we are in the presence of two types of synlophe sharing the presence of a large careen:

Type I (11A–11C’) with careen plus 14 ridges at midbody probably in both sexes; ridges (excluding careen) medium-sized to small, irregularly spaced, with many gaps and 2 medium-sized right ridges with divergent tips. Axis of orientation probably subfrontal.

Type II (11D/11D’), with careen plus 16 ridges within proximal body in female (unknown in male). Ridges (excluding careen) small, subequal and regularly spaced, right ridges very large with parallel tips oriented downwards. Axis of orientation possibly double.

The scales corresponding to the first and third section are the same (25 μm). This would mean that the female body diameter at the distal part is smaller than at the proximal body, which is at least unusual. The figures, from the text, correspond to two females, enlarging the possibility that they come from different taxa.

##### 3.11.2.2 Bursa

The right lobe is in right latero-dorsal view, the left lobe is in left lateral view, the dorsal lobe is in dorsal view. The pattern is 1-4 in both lobes; right lobe with ray 3 diverging first from common trunk, then rays 4-6 at same level; left lobe with rays 3-4 diverging at same level and ray 6 proximally to these.

#### 3.11.3 Conclusion

The specimens described as *Paramelomystrongylus dessetae* seem to be a composite of two different taxa, both belonging to the Nippostrongylinae and possessing synlophes with careen. Type I synlophe evokes the male section of the genus *Helgenema* (Figs. 10B, 10B’) with a weakly developed careen. However, the available data are too limited to conclude that it is the same taxon.

Type II does not resemble any described synlophe and the orientation of the right ridges is unique among the Nippostrongylinae.

Concerning the bursa, we have no elements to attribute the illustrated bursa to a given type of synlophe.

Since we do not know what type of synlophe the holotype corresponds to, *Paramelomystrongylus dessetae* is considered a *species inquirenda*. Being the type species of the genus, it is impossible to give a proper definition of this latter. We thus consider *Paramelomystrongylus* a *genus inquirendum*.

The two types of synlophe recognized seem to be distributed into two different taxa as follows: “*Paramelomystrongylus dessetae*” (male section at midbody and female sections within proximal and distal body) (with synlophe of Type I); Nippostrongylinae *i.s.* 6 (female section at midbody) (with synlophe of Type II).

## 4 Discussion

The revision conducted above led us to consider valid only three of the 11 genera considered: *Melomystrongylus*, *Pogonomystrongylus* and *Nugininema*. The remaining ones: *Mawsonema*, *Montistrongylus*, *Parvinema*, *Missimstrongylus*, *Flannerystrongylus*, *Helgenema* and *Paramelomystrongylus* appear to us insufficiently described or seem to involve more than one taxon; we consider them *genera inquirenda*. With respect to *Rodentanema*, it does not belong to the Nippostrongylinae but to the Herpetostrongylidae (Heligmosomoidea).

In addition to the genera and species considered above, between 2008 and 2021, 26 species of Nippostrongylinae distributed into other genera were described from New Guinean murids (Table 2). The status of most of those genera and species was dealt with in 2014 [2] and 2015 [8]. The taxonomic status or generic attribution of thirteen species described from 2015 to 2021 will be only briefly addressed herein (Table 2). Instead, the biogeographical distribution of the 15 valid genera of Heligmonellidae reported from New Guinean rodents is updated as follows:

### 4.1 Genera only reported from New Guinea

4.1.1 *Lesleyella –* with *Lesleyella wauensis* (Smales, 2010) (= *Odilia wauensis*) in *Lorentzimys nouhuysi* [8, 31].

4.1.2 *Melomystrongylus –* with *M. sepikensis* in *Melomys rufescens* and *Melomys* spp.; and *M*. *somoroensis* in *Paramelomys rubex*.

4.1.3 *Nugininema –* with *N. titokis* in *Rattus niobe*.

4.1.4 *Pogonomystrongylus –* with *P. domaensis* in *Pogonomys loriae*.

4.1.5 *Sanduanensis –* with *Sanduanensis dividua* (Smales, 2014) (= *Odilia dividua*) in *Pogonomys macrourus* [8, 36].

***Odilia helgeni*** Smales, 2015 and ***Odilia whittingtoni*** Smales, 2015, both parasitic in *Pogonomys sylvestris* [37], do not belong to *Odilia*, their synlophe not having a careen. Both species can be related to *Sanduanensis* by characters such as the small number of ridges at midbody (16), but the ventral ridges are continuous *versus* interrupted in *Sanduanensis*. Pending a more precise description of the synlophe of these two species, we consider them as Nippostrongylinae *i.s*.

### 4.2 Genera reported from New Guinea and other islands of Indonesia

4.2.1 *Bunomystrongylus –* with *Bunomystrongylus ilami* Smales, 2015 parasitic in *Pogonomys championi* from New Guinea [37]; plus two species including the type species in *Bunomys* spp. from Sulawesi [13].

*Bunomystrongylus* is mainly characterized by the absence of a careen and the presence of ridges of two types: rounded ridges without cuticular struts – mainly dorsal and left-dorsal – and remaining ridges pointed, with cuticular support. Other characters are the right-dorsal and left-ventral ridges larger, and the female vestibule long and coiled.

The written description and the illustration of *B. ilami* are too brief. Only the male synlophe is illustrated and includes certain anomalies such as, for instance, the unlikely orientation of ridge 1 towards the ventral side, determining (with ridge 1) a pair of divergent ridges on the left side, which is unlikely and through which, in addition, an axis of orientation is drawn. Unlike the other two species in the genus, the right ridge is clearly larger than the left ridges and the adjacent dorsal ridges are the largest.

In addition, species of *Bunomystrongylus* have a very long vestibule. From the written description it would be also the case in *B. ilami* but the illustration is contradictory, the vestibule being extremely short (Fig. 8 in [37]. This species was originally attributed to *Bunomystrongylus* based on one character: the presence of ridges of two types. This character alone seems insufficient and it is not possible to compare appropriately the other generic characters against the other two species. Until improvements are made, even if *B. ilami* seems to be related to *Bunomystrongylus*, it would be preferable to consider it a Nippostrongylinae *i.s.* pending a new complete redescription of the species.

4.2.2 *Hasanuddinia –* with three species from New Guinea: *Hasanuddinia chiruromyos* Smales, 2011 in *Chiruromys vates* [32], *Hasanuddinia pogonomyos* Smales, 2014 and *Hasanuddinia hasegawai* Smales, 2015 both in *Pogonomys sylvestris* [36, 37]; plus the type species from endemic murines from Sulawesi [14]. We agree with Smales [32, 36, 37] in that the three species from New Guinea belong to *Hasanuddinia*, which is characterized by the absence of a careen and the presence of two to three ventral comaretes.

4.2.3 *Hasegawanema –* with two species from New Guinea: *Hasegawanema yuroense* (Smales, 2019) in *Paramelomys platyops* [42] and *Hasegawanema mallomyo*s (Hasegawa & Syafruddin, 1994) in *Mallomys rothschildi* [15]; plus four species (including the type species) in endemic murines from Sulawesi [18]. Species of *Hasegawanema* are characterized by 15–26 ridges including a careen supported by two small ridges of which the ventral one (ridge 1’) may be slightly larger and is distinct from the left ridge. *Hasegawanema yuroense* has 21–23 ridges, but in the illustration of the female synlophe (male not illustrated) the careen is difficult to identify. From the written description, ridge 1’ is distinct from the left ridge but, the lateral fields not being illustrated, this cannot be confirmed. Therefore, this species cannot be assigned to *Hasegawanema*. It seems rather to belong to the group of Australasian genera without careen, but the description is insufficient to place the species in a given genus. We consider *H. yuroense* a Nippostrongylinae *i.s*.

4.2.4 *Hughjonestrongylus –* with 13 species mainly parasites of *Paramelomys* spp. and *Melomys* spp. from New Guinea; plus *Hughjonestrongylus woolleyae* Smales, 2017 parasitic in *Paramelomys lorentzii* from New Guinea and the Aru Islands (Table 2) [29, 30, 32, 40, 42, 43, 45].

Five nominal species plus a *Hughjonestrongylus* sp. were placed in this genus by Digiani & Durette-Desset [2]. We consider that the eight other species described between 2017 and 2020 possess the features of the genus *Hughjonestrongylus*: 20–30 cuticular ridges, careen absent, ridges markedly unequal in size, and mid-left and mid-right ridges largest.

***Odilia carinatae*** Smales, 2008 was described as parasitic in *Uromys* spp. from Papua New Guinea [29]. The female synlophe of this species shows characters of *Hughjonestrongylus*, but the male synlophe does not. Consequently *O. carinatae* was considered Nippostrongylinae *i.s.* by Durette-Desset & Digiani [8].

***Odilia hagemannae*** Smales, 2016 parasitic in *Rattus giluwensis* from New Guinea [39] possesses a synlophe with a careen on the left-dorsal quadrant. This character evokes species of *Maxomystrongylus* Hasegawa and Syafruddin, 1997, parasitic in Muridae (*Maxomys, Rattus and Niviventer*) from Borneo and Sulawesi [16]. The careen of *O. hagemannae* is made up of four thick ridges of which ridge 1’ is thicker than the other ridges. Whereas in the two species of *Maxomystrongylus* the synlophe is made up of only three thin ridges. Without a more complete description of its synlophe (particularly the position of the lateral fields) we prefer to consider *Odilia hagemannae* a Nippostrongylinae *i.s.*

### 4.3 Genera reported from New Guinea and Malaysia

4.3.1 *Macrostrongylus* – with *Macrostrongylus ingens* Smales, 2008 parasitic in various Hydromyinae (*Uromys*, *Melomys*, *Paramelomys* spp.) from New Guinea and the Aru Islands [29, 30, 45]; plus the type species parasitic in *Rattus* spp. from Malaysia [21] (Table 2). Both species of *Macrostrongylus* are very similar and seem to be closely related. The genus was transferred from the Brevistriatinae to the Nippostrongylinae by Durette-Desset *et al*. [10].

4.3.2 *Sabanema* – with *Sabanema macrovulva* Ow–Yang, Durette-Desset & Ohbayashi, 1983 in *Uromys anak* from Papua Indonesia [47]. *Sabanema macrovulva* and the other four species including the type species are mainly parasitic in *Rattus* spp. from Malaysia [21]. The record from *U. anak* is not accompanied by an illustration and consequently it is not possible to confirm the specific attribution of the worms.

### 4.4 Genera reported from New Guinea and Australia

4.4.1 *Equilophos –* with *Equilophos similis* (Smales, 2009) (= *Odilia similis)* parasitic in *Melomys rufescens* from New Guinea [8, 30]; plus *Equilophos polyrhabdote* (Mawson, 1961) parasitic in *Rattus fuscipes assimilis* from Australia [19].

4.4.2 *Parasabanema* ***–*** with two species from New Guinea: *Parasabanema szalayi* Smales & Heinrich, 2010 (type species) and *Parasabanema sene* Smales, 2020, both parasitic in *Paramelomys* spp.; plus *Parasabanema praeputiale* (Gibbons & Spratt, 1995) from Australia [12, 43, 45].

4.4.3 *Chisholmia* Durette-Desset & Digiani, 2015 *–* with *Chisholmia mawsonae* (Durette-Desset, 1969) (=*Odilia mawsonae*), originally described in Australian *Melomys* spp. [3, 48], was reported in *Melomys lutillus* from Papua New Guinea and in *Melomys burtoni* (probably conspecific with *M. lutillus*) from Queensland [26].

4.4.4 *Odilia* Durette-Desset, 1973 with several species:

*Odilia emanuelae* (Mawson, 1961), originally described from the Australian *Rattus fuscipes*, *R. sordidus* [19, 23], *R. leucopus* [46] and *Melomys cervinipes* [48], was reported in New Guinea from *Hyomys dammermani* and *Hyomys goliath* [27], *Pseudohydromys germani* (*= Mayermis ellermani*) and *Parahydromys asper* [28].

*Odilia mackerrasae* (Mawson, 1961), originally described from *Melomys* spp. and *U. caudimaculatus* from Queensland [19, 48], was reported in New Guinea from *Abeomelomys sevia* [35], *Chiruromys vates* [32], *Coccymys ruemmleri* [33], *Melomys lutillus* [26], *M. rufescens* [30], *Parahydromys asper* [28], *Paramelomys rubex* [45], *Pogonomys loriae*, *P. macrourus* [36], *P. sylvestris* [37], and *U. caudimaculatus* [47].

***Odilia melomyos*** (Mawson, 1961), originally described from *Melomys* spp. and *U. caudimaculatus* from Australia [19, 47, 48], was first reported in Papua New Guinea from *Melomys lutillus* (sharing the same helminth community with *Melomys burtoni* from Queensland) [26]; then in *U. anak* and *Uromys caudimaculatus* from New Guinea [47].

***Odilia uromyos*** (Mawson, 1961) parasitic in *Uromys* spp. from Australia [19] was reported from New Guinea in *U. caudimaculatus* and *U. anak* [47].

Since the erection of the genus *Odilia* to encompass a number of species originally described by Mawson [19], it was thought that the genus was only parasitic in Australian Muridae. However, since 1994 several species of *Odilia* started to be described in murids out of Australia, *i.e.* mainly from Indonesia and New Guinea. The first species of *Odilia* reported from New Guinea was *Odilia* sp. parasitic in *Pseudohydromys murinus* [25]. The species was not described or illustrated due to the limited material available, but was stated to differ from all the species in the genus in the spicule length and in the number and relative sizes of the ridges of the synlophe at midbody. This record contributed to the contention that the genus was widely distributed in the region; a contention that was reinforced with the subsequent descriptions of *O. carinatae* and *O. implexa* (Smales 2008) [29]. Several species descriptions followed, and by 2015 the genus *Odilia* was composed of 20 species from Indonesia, Australia and New Guinea, characterized by a great heterogeneity of the synlophe: some species possessed a careen, other species did not, the number of ridges varied from 14 to 35, and there was a notorious disparity in the relative size and development of the ridges, particularly the lateral ones. This brought Durette-Desset & Digiani [8] to undertake a taxonomic revision of the genus, which resulted in the splitting of *Odilia* into eight genera, of which five were defined in that work. Three of those genera (*Hughjonestrongylus*, *Lesleyella* and *Sanduanensis*) were considered endemic to New Guinea, one (*Hasegawanema*) to New Guinea and Sulawesi, two (*Equilophos* and *Parasabanema*) to Australia and New Guinea, whereas *Chisholmia* and *Odilia* were considered endemic to Australia (with one species of *Odilia* from Tasmania). The updated geographical distribution provided in the present work (with the addition of genera and species described between 2008 and 2021), is still largely concordant with that presented in [8].

Among the former species of *Odilia* reported from New Guinea, *O. uromyos* seems to be closely related to *Equilophos* and *Parasabanema* by the high number of continuous ridges observed *in toto*. However, a transverse section of the body would be necessary to determine which genus it belongs to. As the synlophe in cross section is still unknown, the species was considered Nippostrongylinae *i.s.* by Durette-Desset & Digiani [8]. Concerning the reports of *C. mawsonae*, *O. emanuelae*, *O. mackerrasae* and *O. melomyos*, these are not accompanied by illustrations and we are unable to confirm the specific attribution of the worms. Consequently, we still cannot affirm that these species of *Odilia* have travelled, mainly with their *Melomys* hosts between Australia and New Guinea as reported in [26] and [29].

### 4.5 Genera reported from New Guinea, Australia and Indonesia

***Nippostrongylus*** Lane, 1923 is represented in New Guinea by *Nippostrongylus magnus* (Mawson, 1961) parasitic in *Rattus leucopus* and also in the same host from Australia [46], *Nippostrongylus sembeli* Hasegawa & Tarore, 1995 parasitic in *U. caudimaculatus* [34] but originally described from *Rattus xanthurus* from Sulawesi [17]; and the cosmopolitan *Nippostrongylus brasiliensis* (Travassos, 1919) parasitic in *Melomys rufescens* [30, 34].

The genus *Nippostrongylus* was proposed by Durette-Desset *et al.* [9] to be Asiatic in origin, reaching Australia with migrating *Rattus* spp. The finding, in New Guinea, of *N. sembeli* in old endemic hosts and also of *N. magnus* primarily described in old and new endemics from Australia [23, 24], supports an Asiatic origin of the genus, with processes of host switching and speciation following migration, as suggested by Smales [34]. These host relationships and geographic distributions also support host migration across the Torres Strait from Australia to New Guinea as the origin of the presence of *N. magnus* in New Guinea as stated by Smales & Spratt [46]. On the other hand, Smales [34] suggested that the arrival of *N. brasiliensis* in Australia is probably a recent one, with the cosmopolitan invasive species of *Rattus*, and that the record of this species in New Guinea in an old endemic host represents an occasional infection, having the same source.
